# Functional and structural insights into the MRX/MRN complex, a key player in recognition and repair of DNA double-strand breaks

**DOI:** 10.1016/j.csbj.2020.05.013

**Published:** 2020-05-16

**Authors:** Renata Tisi, Jacopo Vertemara, Giuseppe Zampella, Maria Pia Longhese

**Affiliations:** Dipartimento di Biotecnologie and Bioscienze, Università degli Studi di Milano-Bicocca, Milan, Italy

**Keywords:** Double-strand break (DSB), DNA damage, MRX/MRN, Mre11, Rad50, Xrs2/NBS1

## Abstract

Chromosomal DNA double-strand breaks (DSBs) are potentially lethal DNA lesions that pose a significant threat to genome stability and therefore need to be repaired to preserve genome integrity. Eukaryotic cells possess two main mechanisms for repairing DSBs: non-homologous end-joining (NHEJ) and homologous recombination (HR). HR requires that the 5′ terminated strands at both DNA ends are nucleolytically degraded by a concerted action of nucleases in a process termed DNA-end resection. This degradation leads to the formation of 3′-ended single-stranded DNA (ssDNA) ends that are essential to use homologous DNA sequences for repair. The evolutionarily conserved Mre11-Rad50-Xrs2/NBS1 complex (MRX/MRN) has enzymatic and structural activities to initiate DSB resection and to maintain the DSB ends tethered to each other for their repair. Furthermore, it is required to recruit and activate the protein kinase Tel1/ATM, which plays a key role in DSB signaling. All these functions depend on ATP-regulated DNA binding and nucleolytic activities of the complex. Several structures have been obtained in recent years for Mre11 and Rad50 subunits from archaea, and a few from the bacterial and eukaryotic orthologs. Nevertheless, the mechanism of activation of this protein complex is yet to be fully elucidated. In this review, we focused on recent biophysical and structural insights on the MRX complex and their interplay.

## Graphical abstract

**Figure:**
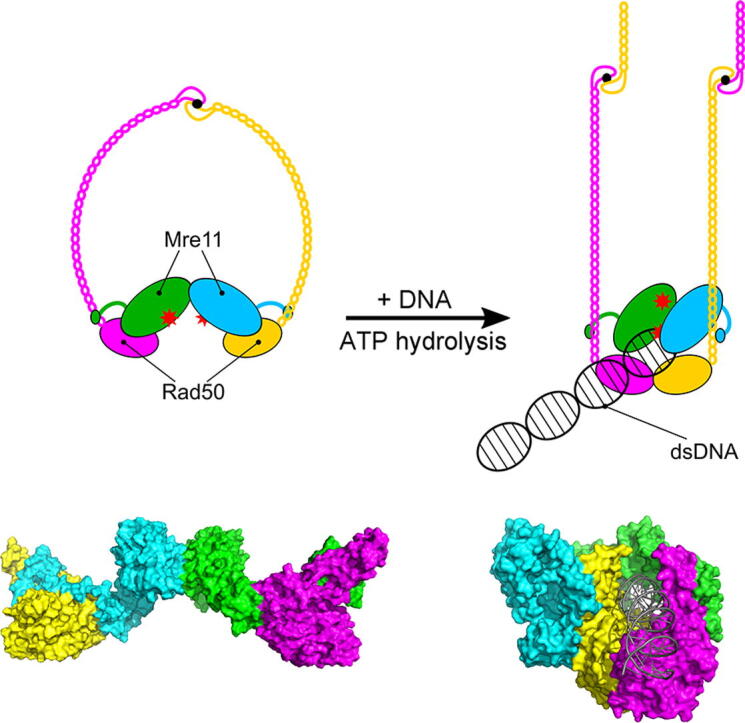

## 1 Introduction

Chromosomal DNA double-strand breaks (DSBs) pose a significant threat to cell viability and genome stability because failure to repair them can lead to loss of genetic information and chromosome rearrangements. Although DSBs threaten genome integrity, germ cells deliberately sever both strands of their chromosomes to initiate meiotic recombination that ensures proper homologous chromosome segregation [1]. Furthermore, DSBs are programmed recombination intermediates during antigen-receptor diversity in lymphocyte development [2].

**Fig. 1:**
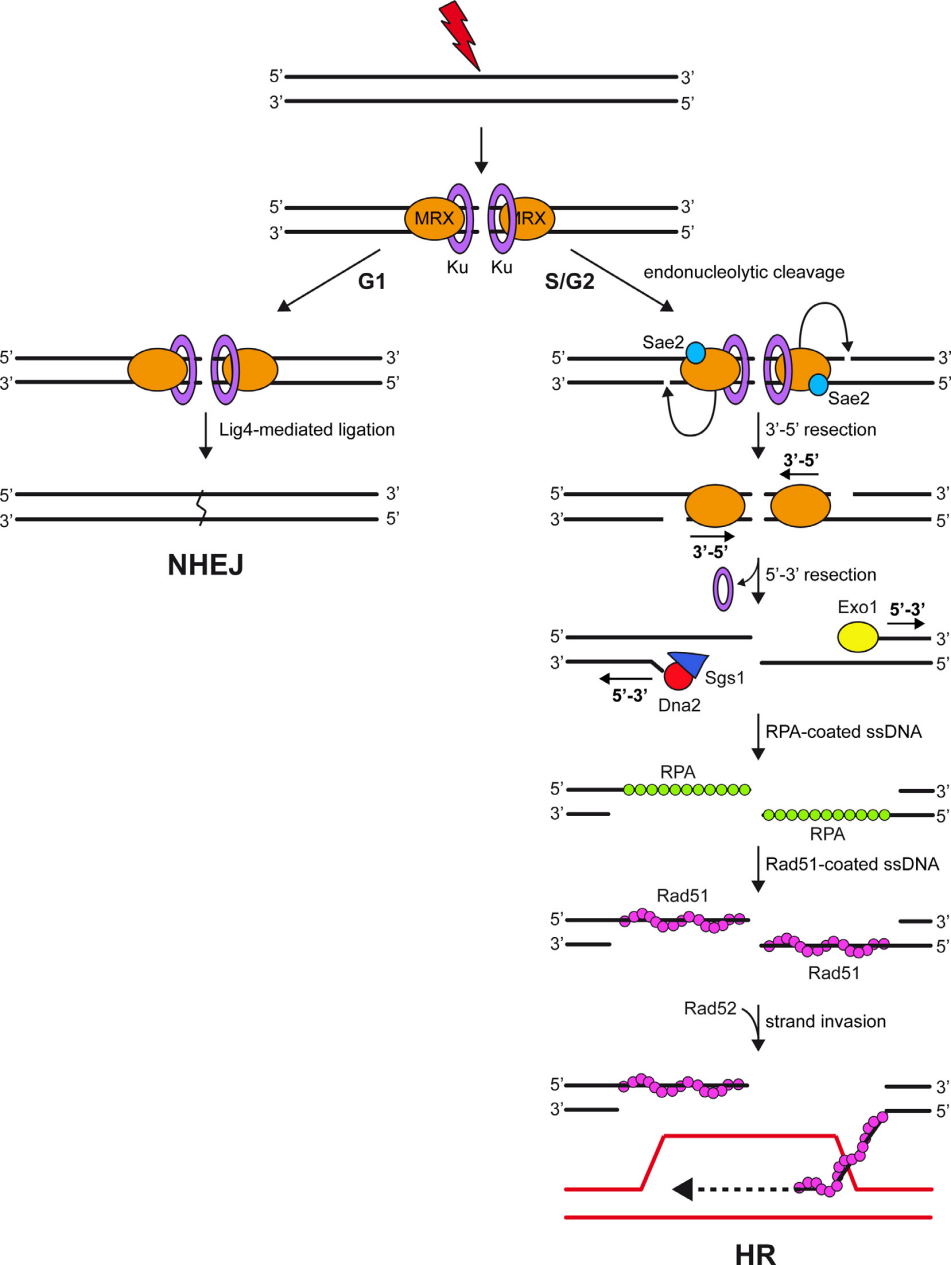
Overview of the DSB repair mechanisms. DSBs are repaired by non-homologous end joining (NHEJ) or homologous recombination (HR). NHEJ directly rejoins the two DSB ends and occurs predominantly in the G1 phase of the cell cycle. HR utilizes homologous template and is active in S and G2. Once a DSB occurs, MRX and Ku are rapidly recruited to the DSB ends. After ATP hydrolysis by Rad50, Mre11 together with phosphorylated Sae2 catalyzes an endonucleolytic cleavage of the 5′-terminated strands at both DSB ends, followed by bidirectional resection catalyzed by Mre11 in the 3′ to 5′ direction and by Exo1 and Dna2-Sgs1 in the 5′ to 3′ direction. RPA binds to the 3′-ended ssDNA overhangs and is then replaced by Rad51. The Rad51-ssDNA complex promotes the homology search and strand invasion, pairing the invading 3′ end with one strand of the donor duplex to template DNA synthesis (dashed line).

Eukaryotic cells have evolved two main mechanisms to repair DSBs: non-homologous end joining (NHEJ) and homologous recombination (HR) (Fig. 1). NHEJ allows direct rejoining of the broken DNA ends with no or minimal base pairing at the junction and it operates predominantly in the G1 phase of the cell cycle [3]. By contrast, HR is the predominant repair pathway in the S and G2 phases of the cell cycle and it requires an undamaged homologous sequence (sister chromatids or homologous chromosomes) to serve as a template for repair of the broken DSB ends (Fig. 1) [4].

The initial step of NHEJ involves the binding to the DSB ends of the Ku70-Ku80 (Ku) heterodimer, which protects DNA ends from degradation and recruits additional NHEJ components such as the DNA ligase IV (Fig. 1) [3]. While NHEJ does not require extensive DSB end processing, HR initiates by nucleolytic degradation of the 5′-terminated strands at both DSB ends, in a process referred to as resection (Fig. 1) [5]. This degradation results in the generation of 3′-ended single-stranded DNA (ssDNA) ends, which are initially bound by the ssDNA binding protein complex Replication Protein A (RPA). RPA is then displaced by Rad51 to form a right-handed Rad51-ssDNA nucleoprotein filament that is essential for the homology search and pairing of the ssDNA with the complementary strand of the donor duplex DNA (Fig. 1) [4]. The 3′-terminated strand at the other side of the break anneals to the displaced strand. Extension by DNA synthesis and ligation generate a double Holliday junction that can be dissolved or resolved to yield intact duplex DNA molecules [4].

One of the primary protein complexes responsible for recognition, signaling and repair of DNA DSBs is the evolutionarily conserved Mre11-Rad50-Xrs2/NBS1 complex (MRX in *Saccharomyces cerevisiae*, MRN in humans). MRX/MRN is rapidly recruited to DSBs, where it initiates DSB resection and maintains the DSB ends tethered to each other for their repair [6]. Furthermore, it is required to recruit and activate the protein kinase Tel1 (ATM in mammals), which coordinates DSB repair with cell cycle progression [7]. Both MRX and Tel1 are also necessary to maintain the length of telomeres, specialized nucleoprotein complexes at the ends of eukaryotic chromosomes [8]. Finally, MRX/MRN also supports DNA replication under stress conditions. In particular, it promotes the recombinational repair of damaged replication forks by resecting nascent DNA strands [9], [10]. In mammals, emerging evidence indicates that MRE11‐mediated degradation of stalled replication forks is restrained by the recombination proteins BRCA1 and BRCA2 that promote formation of stable RAD51 nucleoprotein filaments [9], [10].

At the molecular level, MRX/MRN is a hetero-hexameric (M_2_R_2_X_2_/N_2_) protein complex, in which the Mre11 subunit dimerizes and interacts independently with both Rad50 and Xrs2/NBS1 [6]. Rad50 is a member of the structural maintenance of chromosomes (SMC) protein family, characterized by ATPase motifs at the N- and C-termini separated by two long coiled-coil domains. The coiled coils fold back on themselves to form two complete ATPase sites on a Rad50 dimer [11], [12].

Mre11 displays 3′–5′ exonuclease and endonuclease activities [13], [14], [15], [16], [17], [18]. In both yeast and mammals, Mre11 catalyzes the endonucleolytic cleavage of the 5′-terminated DNA strand in the vicinity of the DSB end [19], [20], [21], [22] (Fig. 1). This endonucleolytic cleavage requires the ATPase activity of Rad50, as well as the Sae2 protein (Ctp1 in *Schizosaccharomyces pombe*, CtIP in mammals) that promotes the Mre11 endonuclease activity within the MRX/MRN complex [23], [24], [25]. The MRX-Sae2 initial cleavage is followed by bidirectional resection using the Mre11 3′–5′ exonuclease, which proceeds back towards the DSB ends, and the nuclease activities of Exo1 (EXO1 in mammals), or of Dna2 (DNA2 in mammals) in complex with the RecQ-helicase homolog Sgs1 (BLM or WRN in mammals), which degrade DNA in the 5′–3′ direction away from the DSB ends [26], [27], [28], [29], [30], [31], [32] (Fig. 1).

While orthologs of Rad50 and Mre11 are found in all kingdoms of life, the Xrs2/NBS1 subunit is specific to eukaryotes. In humans, germline hypomorphic mutations in MRE11, NBS1, or RAD50 are associated with ataxia telangiectasia-like disorder (ATLD), Nijmegen breakage syndrome (NBS), and NBS-like disorder (NBSLD), respectively, which are characterized by cellular radiosensitivity, immune deficiency and cancer predisposition [33]. Aside from germline mutations, all three MRN complex components are mutated in more than 50 types of cancer, as assessed from the International Cancer Genome Consortium projects. Furthermore, in mammals, the MRN complex is essential for cell viability, as deletions of any MRN subunits result in embryonic lethality [34].

## 2 MR complex architecture

Recent findings have added more and more elements to the complexity of MRX/MRN flexible and dynamic mechanism of action, which evolved in order to integrate protein–protein and protein-DNA interactions, together with Mre11 intrinsic enzymatic activities, with organism specific regulatory networks. A plethora of structural and biophysical data were added to genetic and biochemical characterization of MRX/MRN function, allowing to develop a generally accepted model for Mre11 and Rad50 (MR) heterotetramer assembly, ATP hydrolysis by Rad50 and Mre11 nucleolytic activities [35]. A couple of decades of analyses by X-ray crystallography, small-angle X-ray scattering (SAXS), analytical ultracentrifugation, inductively coupled plasma mass spectrometry, dynamic light scattering, atomic force microscopy (AFM), electron microscopy (EM) and lately cryo-electron microscopy (cryo-EM) were collected and generally agreed on a well conserved architecture for a tetrameric complex constituted by a dimer of dimers of Mre11 and Rad50 (usually indicated as M_2_R_2_).

**Fig. 2:**
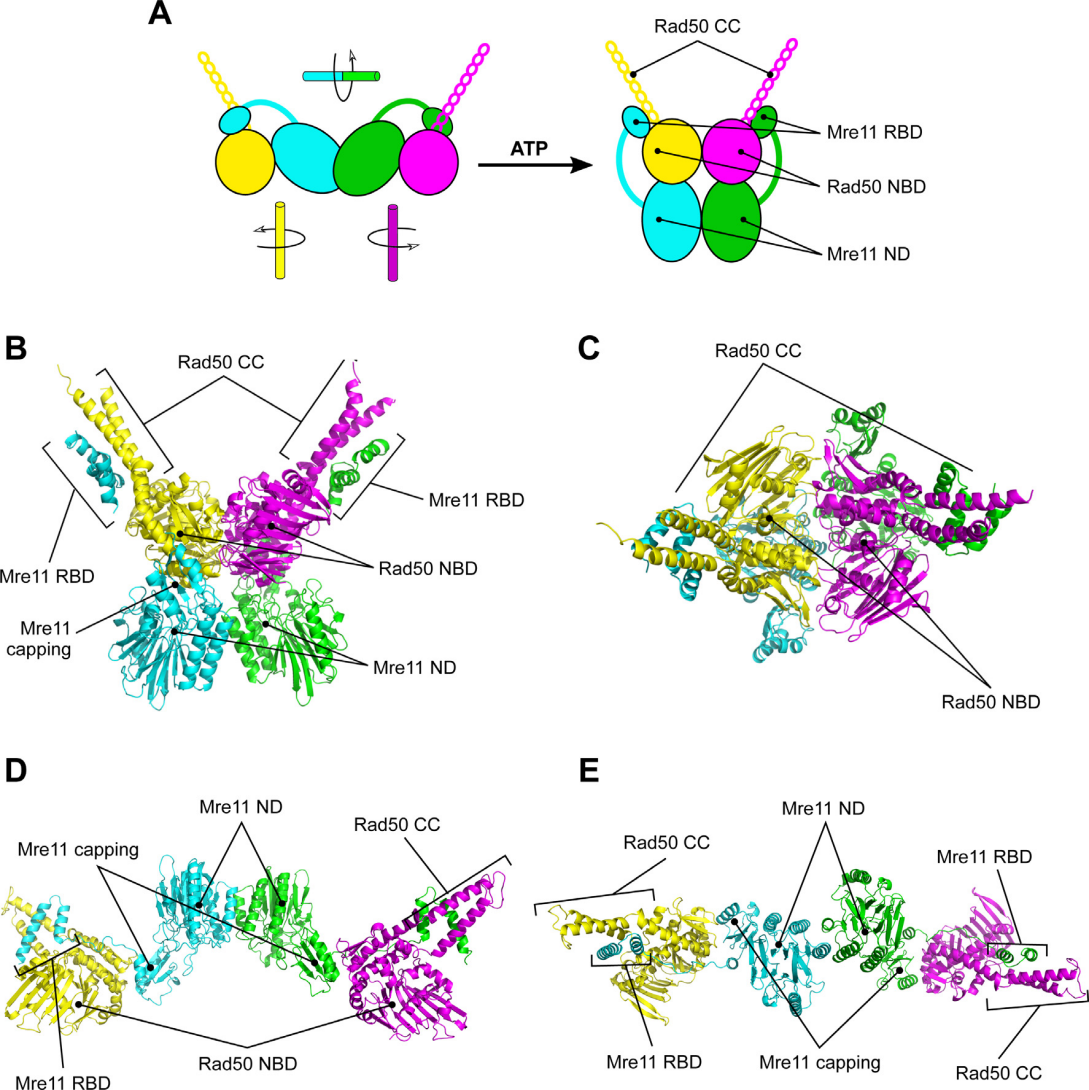
The Mre11-Rad50 heterotetrameric complex can assume different configurations depending on nucleotide binding. (A) Schematic representation of the ATP-bound and nucleotide-free M_2_R_2_ complex configurations as characterized by SAXS. (B, C) Structure of the archaean *Methanocaldococcus jannaschii* Mre11-Rad50 heterotetrameric (M_2_R_2_) complex in presence of an ATP analogue (closed configuration) (B, front view; C, top view; PDB ID: 5DNY). (D, E) Structure of the bacterium *T. maritima* M_2_R_2_ complex in the absence of ATP (open configuration) (D, front view; E, top view; PDB ID: 3QG5). RBD, Rad50 binding domain; NBD, nucleotide binding domain; ND, nuclease domain; CC, coiled coil. Mre11 subunits are represented in green and cyan; Rad50 subunits are in magenta and yellow. (For interpretation of the references to colour in this figure legend, the reader is referred to the web version of this article.)

Early EM and AFM analyses had revealed a structure characterized by a globular head and a long, straight rod or ring bent projection that can adopt different conformations [11], [36], [37], [38], [39], [40], [41]. Structural studies allowed to identify the head as deriving from the association of two Rad50 nucleotide binding domains (NBD) and two Mre11 nuclease domains (ND), while the projections are constituted by the about 500 Å anti-parallel coiled-coil (CC) domain in the middle of Rad50 molecule (Fig. 2A) [11], [12]. The coiled coils can form large proteinaceous rings or rods, which are joined by a CXXC motif at the apex of the coiled-coils that mediates Rad50 subunits interactions via tetrahedral coordination of a zinc ion [37], [39], [42], [43], [44], [45], [46], [47], [48].

Rad50 NBD contains the ABC ATPase domain, comprising the N-terminal Walker A and the C-terminal Walker B, as well as signature motifs typical of this family (Fig. S1A-C). Binding of a non-hydrolysable ATP analogue (AMP-PNP) to Rad50 induces a dramatic conformational rotation of the C-terminal ATPase subdomains (lobe II) relative to the N-terminal half (lobe I) [11], [49], [50], [51] that increases the binding affinity of two Rad50 subunits [49], [51]. This rotation also allows the formation of a dimer, which adopts a closed conformation with two molecules of ATP shared at the interface (Fig. 2B and C) [49], [51].

Mre11 has five highly conserved phosphodiesterase motifs in the N-terminal nuclease domain (Fig. S2A and B) [13], [14], [15], [16], [17], [18]. Besides, Mre11 protein is composed by a capping domain and a Rad50 binding domain (RBD). RBD consists in a helix-loop-helix (HLH) domain that takes contact with the base of Rad50 CC portion [50], [51], [52] (Fig. 2B and C). Actually, Mre11 embraces Rad50-ATP dimer not only with its RBD, but also with residues in the nuclease and the capping domains as well, resulting in Mre11 being completely inaccessible to double-stranded DNA (dsDNA) [50], [51], [52], [53], [54], [55], [56] (Fig. 2B and C). Residues in the nucleolytic catalytic sites are also involved in stabilizing protein–protein interaction [57]. The N-terminal and C-terminal portions of Mre11 are structurally and functionally distinct [58] and connected by a long and largely disordered linker that ensures high flexibility.

In the absence of ATP, the MR complex was resolved in a configuration where Mre11 only holds the Rad50 ATPase domains near the base of the coiled coils by its RBDs [50], while the NBDs of Rad50 are wide open and available to contact dsDNA (Fig. 2D and E). Other interfaces were proposed to stabilize this open configuration and involve Mre11 capping domain competing with ATP for Rad50 signature motif binding [50]. Mutations preventing this predicted second interface to be settled (such as *S. cerevisiae* Y328A) actually destabilize Rad50 dimer association with Mre11 and partially or completely fail to rescue the sensitivity to DNA damaging agents of *mre11*Δ cells [50], suggesting that this configuration could be a transition state required for correct MR assembly (see Table S1 for complete mutants information).

At the top of the long Rad50 CC domain, conserved and essential CXXC motifs allow formation of two zinc-hooks that are critical for MRX complex function, and whose conformation influences the structural behavior of the head domains [39], [44], [59], [60], [61]. Furthermore, an additional interface is present within the *Homo sapiens* Rad50 distal coiled-coil domains, also validated in a corresponding *S. cerevisiae* hypomorphic *rad50-48* mutant, which is defective in Rad50 dimerization due to loss of CC stabilization of the hook [48].

**Fig. 3:**
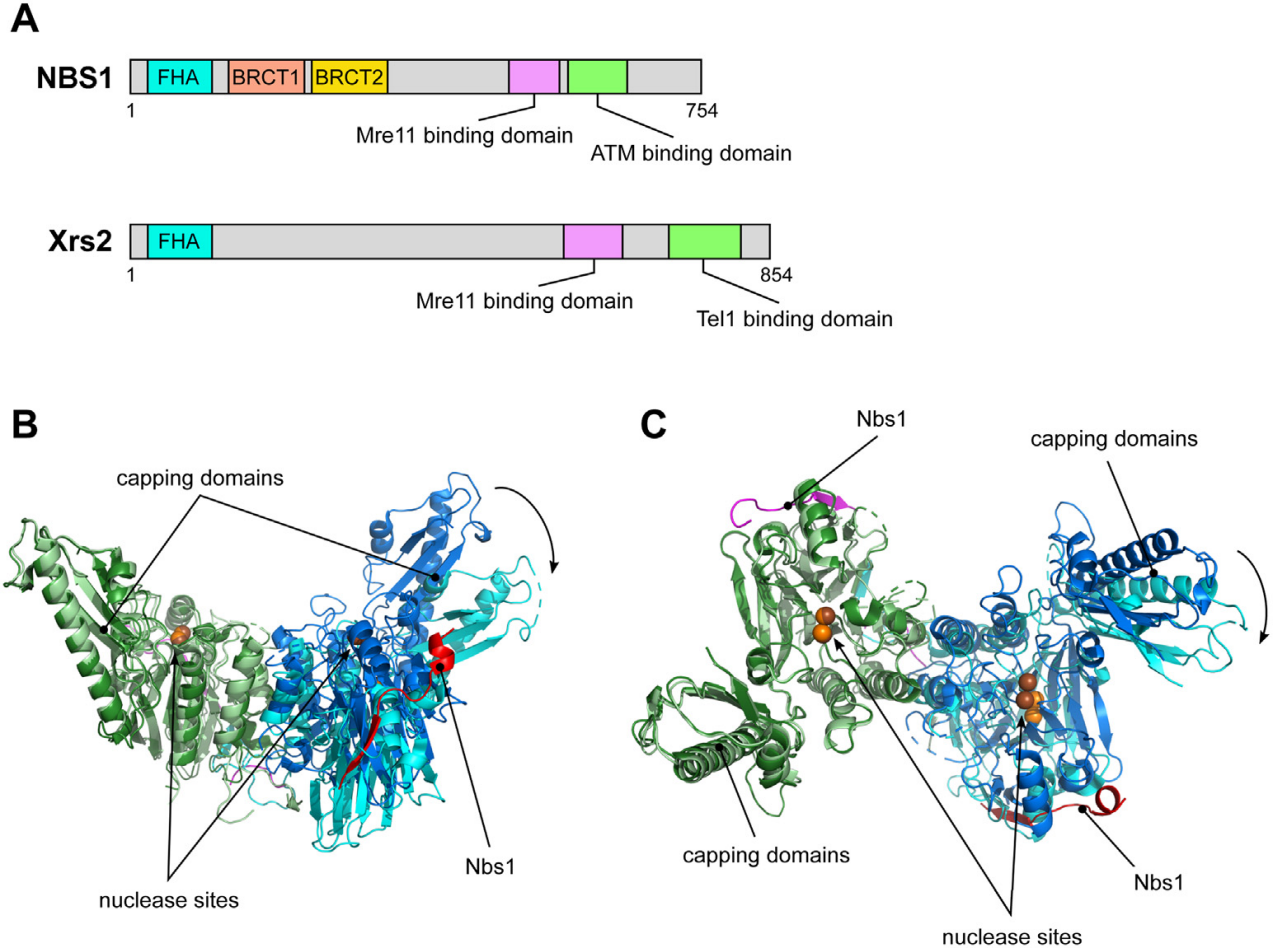
Effect of Nbs1 binding on *S. pombe* Mre11 dimer conformation. (A) Scheme depicting the conserved domains present in NBS1 and Xrs2. (B, C) Mre11 dimer from *S. pombe* (PDB ID:4FCX) either alone or with an associated fragment of Nbs1 (aa 474–531) (PDB ID:4FBW). Mre11 subunits are represented in dark blue and green; Nbs1 is in red. (For interpretation of the references to colour in this figure legend, the reader is referred to the web version of this article.)

The third component of MRX/MRN complexes, Xrs2/NBS1, is far less conserved than the previous ones. Apart from the functional similarities, Xrs2 and NBS1 have different structural and functional features (Fig. 3A). They both show a fork-head associated (FHA) domain in the N-terminal and Mre11 and Tel1/ATM interaction domains, as well as nuclear localization signals in the C-terminal that promote the nuclear import of MRN/MRX [62], [63], [64], [65], [66], [67]. In *S. pombe*, Nbs1 was revealed to wrap as an extended chain around the Mre11 phosphodiesterase domain with 2:2 (M:N) stoichiometry, but only one of the two Nbs1 completely binds via the NFKXFXK motif to the Mre11 latching loop (Fig. 3B). The stability of this last interaction was reported to be fundamental for at least some of MRX/MRN complex functions, which are compromised when amino acid substitutions corresponding to ATLD/NBSLD clinical mutations were introduced in *S. pombe* Mre11 (for example N113S) [68]. It is still unclear if this asymmetric bridging of the Mre11 dimer has any functional meaning: DNA-bound archaeal and *S. pombe* Mre11 structures have different angles in the Mre11 dimer, probably due to the difference in the dimerization interface and thanks to the presence of the latching loop only in eukaryotic orthologs (Fig. S2B). High flexibility of the latching loop confers dynamic properties to eukaryotic dimers suggesting that conformational changes in the Mre11 dimer due to Mre11-Nbs1 interaction could be relevant for MRN function (Fig. 3B). In detail, a variation in Mre11 dimer angle rotation, controlled by Nbs1 on one side and by DNA and/or Rad50 plus ATP on the opposing side of Mre11, might be sensed by effectors of the complex and/or directly influence nuclease activity. Nevertheless, the dimer interface residues of bacterial and archaeal Mre11 also undergo conformational changes upon Rad50 dependent ATP binding [13], [50], [51], [56].

While Xrs2 is largely disordered, NBS1 also contains two BRCT (Breast Cancer Suppressor Protein BRCA1) domains in the N-terminal, after the FHA (Fig. 3A), and they are all involved in the recruitment of NBS1 to phosphorylated histone H2AX at DSBs [69], [70], [71], [72], [73], [74]. NBS1 was also recently reported to sense CtIP phosphorylation with its N-terminal domains and activate MRE11 endonuclease activity [75], but this feature does not seem to be shared by Xrs2. In fact, the N-terminal FHA domain of *S. cerevisiae* Xrs2 binds phosphorylated Sae2, although this capacity appears to be partially dispensable for DSB end resection [76].

## 3 ATP hydrolysis drives a huge MR complex reconfiguration

Several structural data suggested that Rad50-catalyzed ATP hydrolysis would induce a switch between a closed state, in which Mre11 nuclease domain is occluded, to an open configuration with exposed Mre11 nuclease sites [50], [51], [55], [56], suggesting that this event would be fundamental for the regulation of MR activity and DNA repair. Structural studies on *P. furiosus* (*Pf*) nucleotide‐free Rad50 revealed a solvent‐accessible channel extending deep into the Rad50 hydrophobic protein core. This channel is substantially remodeled and reduced when the ATPase subdomain rotates in response to ATP binding by concerted movement of R805 and R797 residues, which block and partially fill the remaining cavity upon ATP binding [51].

Structures of Rad50 alternative conformational states were resolved in different organisms [11], [49], [50], [51]. Some insights, obtained by methyl-based NMR spectroscopy [77] and by molecular dynamics [78], suggest that conformational changes in the α1-β4 loop in Rad50 would be involved in the molecular events driving Rad50 transition from the ATP-bound to the ADP-bound state. Consistently, a shift in α1-β4 loop conformation was previously reported for the *Pf* Rad50^R805E^ mutant variant, which is characterized by high affinity ATP binding and slow hydrolysis rate [55]. Upon ATP-binding, the switch of Rad50 R12 residue towards the nucleotide releases K54 in the α1-β4 loop, which moves to the protein surface gaining solvent accessibility. The R805E mutation induces a conformational rearrangement that alters a neighboring hydrogen bonding network of Y157, R12, and the α1-β4 loop residues D41, D60 and K54, releasing this last and facilitating the transition to the ATP-bound state. Interestingly, deletion of the entire α1-β4 loop (Δ51–60) eliminates ATP‐stimulated DNA binding of full‐length MR [55].

Rad50 ATPase activity is intrinsically low and it is not clear yet which molecular event triggers ATP hydrolysis, although the coiled-coils and hook domains were proposed to influence the catalytic behavior of Rad50 NBD by a long-range allosteric mechanism [48]. Consistently, single amino acid substitutions (e.g. L828F and D829N) in a conserved motif in the D loop (Fig. S1C) of Rad50 NBD were reported to increase the ATP hydrolysis rate, particularly when they were coupled with CC domain shortening [79]. These mutant variants seem to adopt a hydrolysis competent state in Rad50 dimer head more easily, which would require the two Rad50 NBDs within the dimer at a wider distance, while the wild-type dwells upon a tightly closed conformation that would be recalcitrant to ATP hydrolysis [79]. Surprisingly, these mutants both in *S. cerevisiae* and in mouse embryonic fibroblast cells show a disruption of the DNA damage response, coupled to a loss of Tel1/ATM kinase activation [80]. This behavior was proposed to rely on the scarce persistence of the ATP-bound MRX configuration. Actually, proper Tel1/ATM activation was recently found to require MRX in a tightly closed configuration [81], also according to molecular dynamics simulation of a Rad50 A78T mutation in budding yeast [78]. The Rad50^A78T^ mutant variant is in fact defective in Tel1 activation and it was found to induce a spontaneous switch in Rad50 conformation, albeit bound to ATP, in molecular dynamics simulation of the MR heterotetramer.

In its turn, ATP hydrolysis triggers large changes in MR configuration that lead to the dislocation of Rad50 from the nuclease domain of Mre11 to clear its nuclease site [50], [51], [53], [54], [55]. The C-linker flexibility is mandatory to allow this reorganization in MR complex. In fact, a Ser499 substitution with the more rigid proline residue in the linker of *S. cerevisiae* Mre11 was reported to affect Mre11-Rad50 interaction, although it is not directly involved in any of the described interfaces [78].

The open configuration would allow M_2_R_2_ complex to contact dsDNA, but the complex would efficiently bind to DSB end only in presence of ATP, suggesting a model where the complex clamps on DNA by adopting the closed configuration [50] (Fig. 2B and C). This model tallies with previous observation by AFM, suggesting that a transition in the orientation of the coiled-coils is dependent on DNA binding by the MRN head [82] and with nucleotide-dependent conformational changes reported at the proximal traits of the coiled-coils in different Rad50 structures [50], [51], [53], [54], [56]. Supporting this idea, the affinity of Rad50 to ATP and its hydrolysis are different when coiled-coils are truncated [55]. The conformational change would require both ATP molecules to be either hydrolyzed or recruited in order to promote dimer dissociation or association, which is ensured by the observed cooperativity of ATP hydrolysis and binding which is typical of ABC-ATPases [40].

**Fig. 4:**
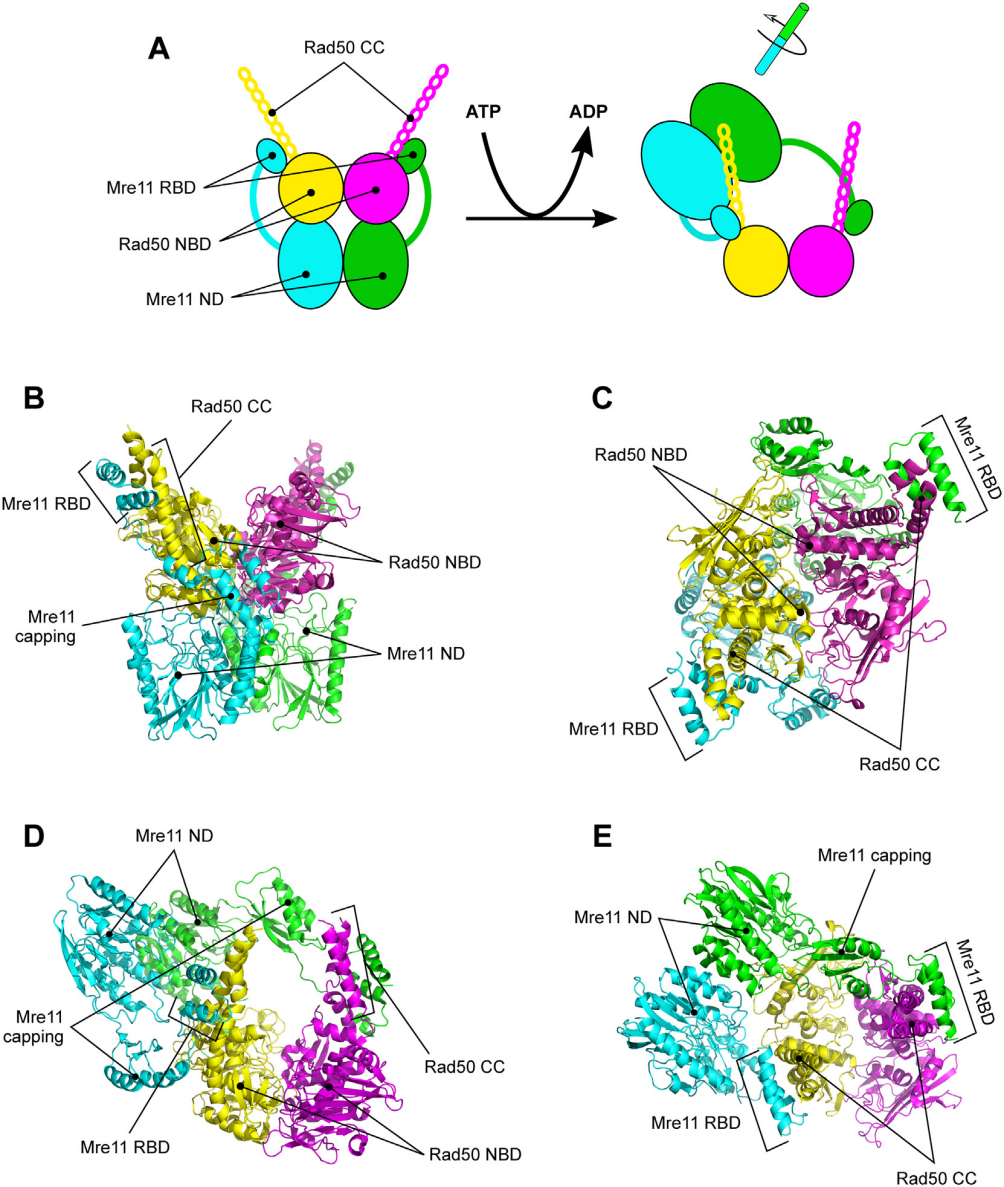
The Mre11-Rad50 heterotetrameric complex can assume different configurations depending on dsDNA end binding. (A) Schematic representation of the ATP- and DNA-bound *E. coli* M_2_R_2_ complex configurations as characterized by cryo-EM. (B, C) Structure of bacterial *E. coli* M_2_R_2_ complex in presence of ATP (closed configuration) (B, front view; C, top view; PDB ID: 6S6V). (D, E) Structure of bacterial *E. coli* M_2_R_2_ complex in presence of ADP and a 60 bp-dsDNA molecule (cutting state configuration) (D, front view; E, top view; PDB ID: 6S85). RBD, Rad50 binding domain; NBD, nucleotide binding domain; ND, nuclease domain; CC, coiled coil. Mre11 subunits are represented in green and cyan; Rad50 subunits are in magenta and yellow. (For interpretation of the references to colour in this figure legend, the reader is referred to the web version of this article.)

**Fig. 5:**
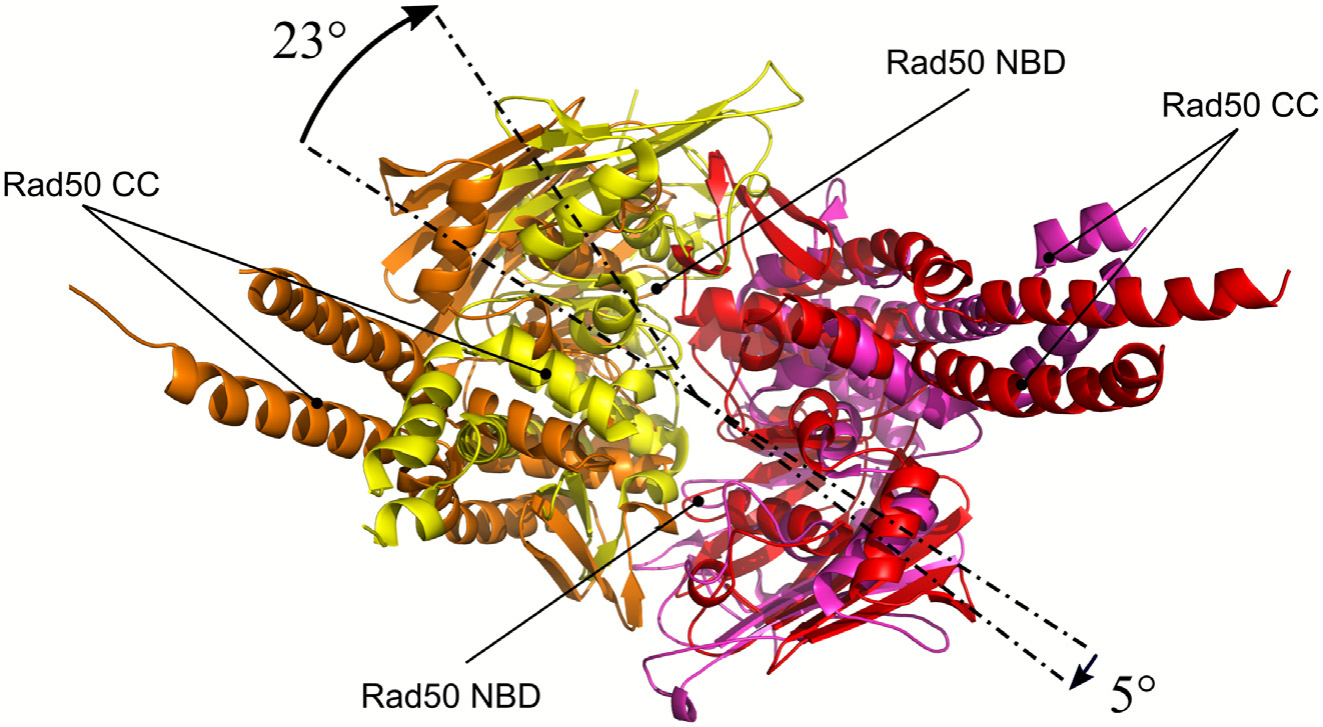
Differences in Rad50-Mre11 dimer of dimers interaction by comparison between heterotetrameric ATP-bound MR structures. Complexes superposition of the structures of M_2_R_2_ complex from *M. jannaschii* (PDB ID: 5DNY, red and orange structures) and *E. coli* (PDB ID: 6S6V, yellow and magenta structures) was obtained by structural alignment of Mre11 dimers. Then, only Rad50 dimers were shown, revealing a non-perfect alignment of the Rad50 dimers in the complexes: two subunits are pivoted by 23° (orange and yellow), while the others are rotated by 5° (red and magenta). (For interpretation of the references to colour in this figure legend, the reader is referred to the web version of this article.)

The early proposed model for dsDNA binding to M_2_R_2_ [50] has striking analogy to a recently resolved structural model (Fig. 4A) based on cryo-EM analysis of SbcC-SbcD, *Escherichia coli* MR, complex either in presence of ATP (Fig. 4B and C) or of ADP and a short dsDNA (Fig. 4D and E) [83]. In the absence of DNA, the *E. coli* tetramer M_2_R_2_ (Fig. 4B and C) adopts a similar but not identical configuration to the previously described ‘closed’ state (Fig. 2B and C). Notably, the orientation of the Mre11 dimer with regards to the Rad50 dimer is not exactly identical to the structures obtained by SAXS on crystals (Fig. 5), though this could be attributable to the different organism of origin (archaea vs prokaryotic origin), to the artificial shortening of the coiled-coil traits in the archaea Rad50 or to the different techniques used to obtain the structure (SAXS vs cryo-EM).

**Fig. 6:**
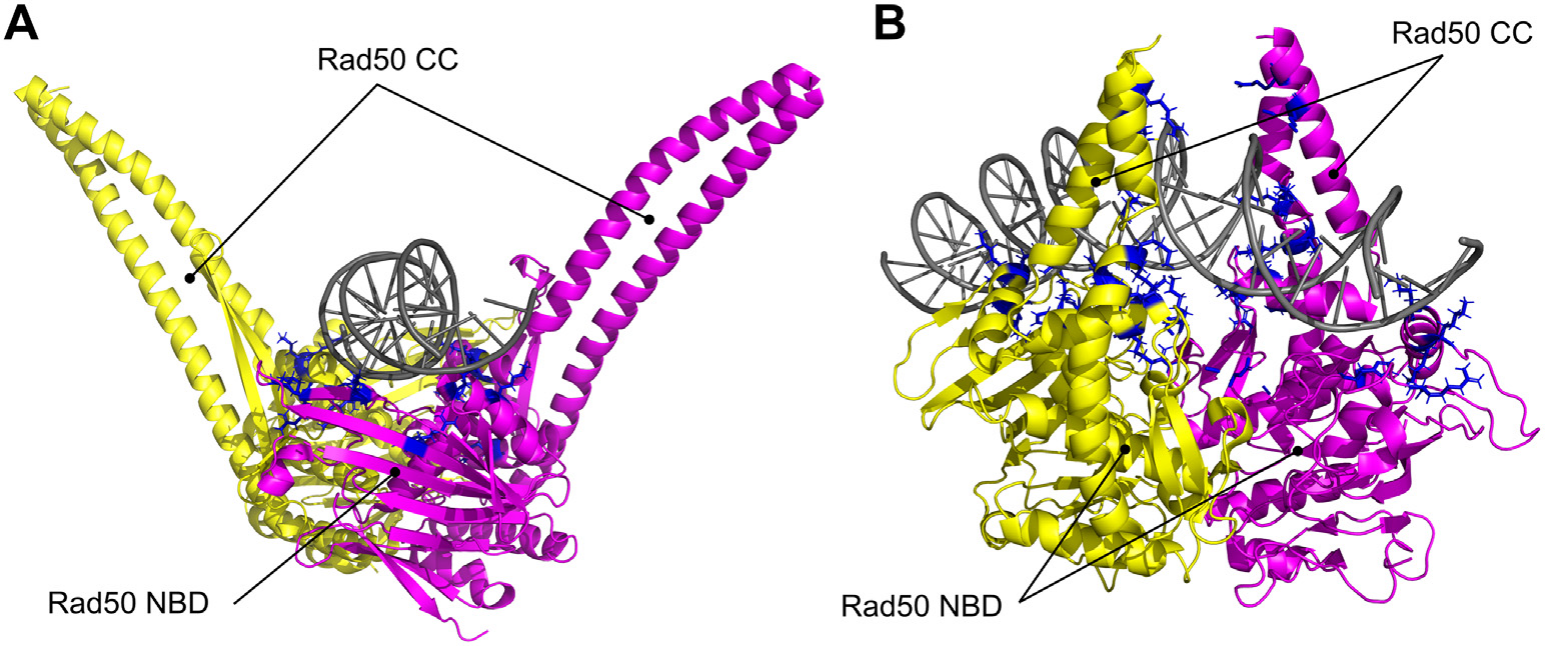
Rad50 dimer can interact in different configurations with dsDNA. The dsDNA-Rad50 interface in the structure of the filamentous fungus *Chaetomium thermophilum* Rad50 dimer in presence of an ATP analogue and dsDNA (A, PDB ID: 5DAC) and in the structure of *E. coli* M_2_R_2_ complex in presence of ADP and dsDNA (B, PDB ID: 6S85) reveals that Rad50 can interact with dsDNA with two different interfaces: Rad50 nucleotide binding domain (NBD) groove and proximal coiled-coil (CC) domain. Rad50 subunits are represented in magenta and yellow; dsDNA is in grey. Basic amino acids (Lys, Arg and His) that can be implicated in DNA binding are shown as blue sticks. (For interpretation of the references to colour in this figure legend, the reader is referred to the web version of this article.)

Upon DNA binding, *E. coli* MR complex actually forms a clamp around dsDNA through the two complete coiled-coils (CC) (Fig. 4D and E). Though the inability of CC to directly contact dsDNA in previous structural models may be due to the artificial truncation (Fig. 6A), here they zip up into a rod and tightly embrace DNA, together with the Rad50 nucleotide-binding domains (Fig. 6B). Consistently with the most recent finding, one previously reported structure of *Thermotoga maritima* Rad50 dimer together with dsDNA revealed a contact between dsDNA and a single Rad50 subunit strand‐loop‐helix motif in the proximal CC [84]. However, corresponding mutations in yeast that abolished this contact were found not to affect DNA double‐strand break repair [84].

**Fig. 7:**
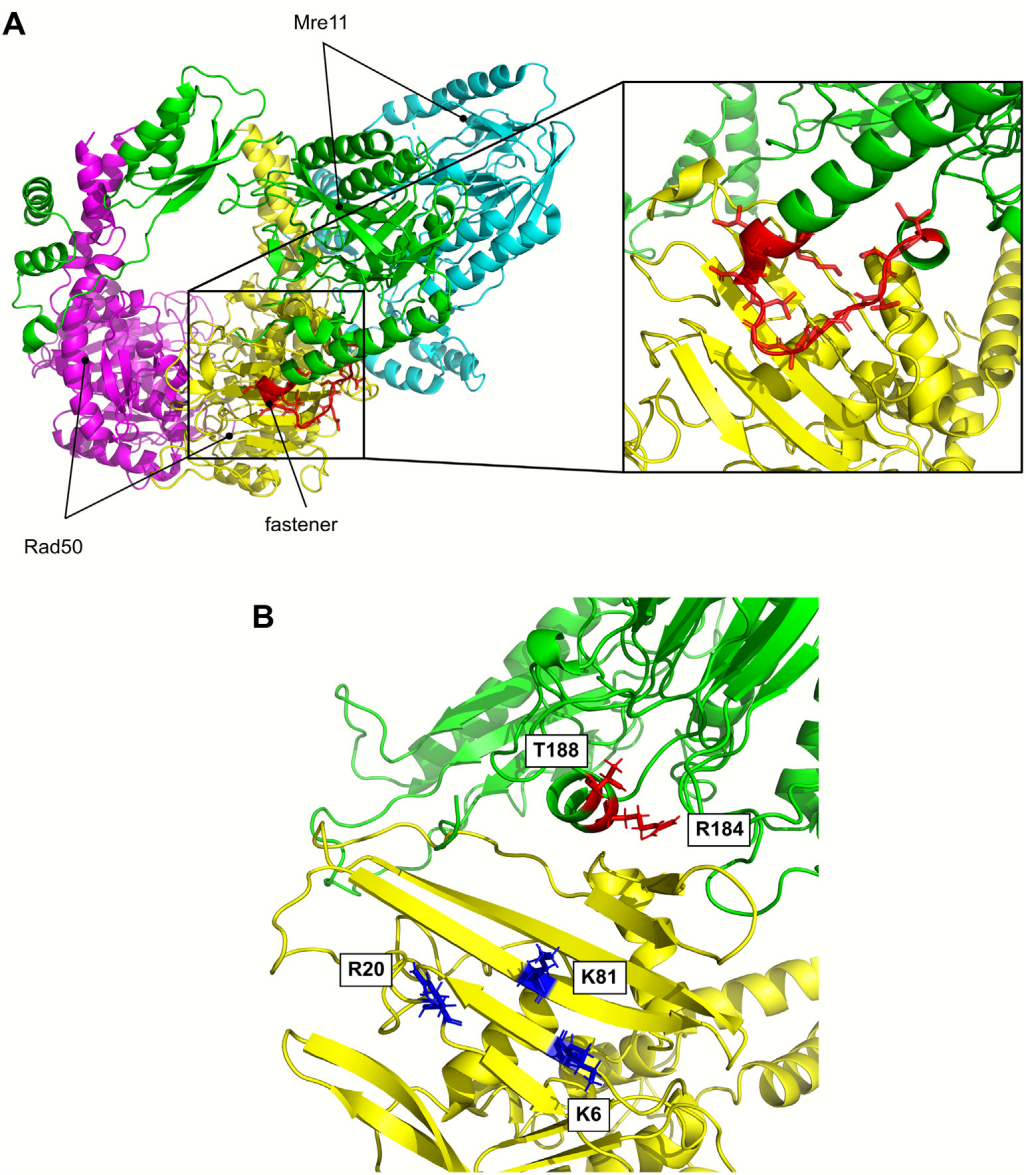
The interface between Rad50 and Mre11 fastener loop in bacterial cutting state of MR complex is not conserved in eukaryotes, but residues involved in Sae2 interaction with MR complex are localized in these regions. (A) Detail of the Mre11-Rad50 interface in the structure of *E. coli* M_2_R_2_ complex bound to DNA in the so called ‘cutting state’ (PDB ID: 6S85). (B) Localization of residues affected by the *rad50S* (K6, R20 and K81), *mre11S* (T188) and R184A mutations in *S. cerevisiae*. Mre11 subunits are shown in green and cyan; Rad50 subunits are in magenta and yellow; fastener loop is in red. (For interpretation of the references to colour in this figure legend, the reader is referred to the web version of this article.)

Upon DNA end binding, Mre11 dimer surprisingly moves to the side of Rad50 dimer, thus reaching the DNA end, which plunges in a channel bordered by Rad50 CC and Mre11 capping domain, and leading to Mre11 nuclease site (Fig. 4D and E). In this configuration, Mre11 and Rad50 share the usual interface between the former RBD and the latter CC domain. A second interface is shared by one of the Rad50 subunits and one of the Mre11 subunits (Fig. 7A), involving in particular the outer β sheet of Rad50 and the 137–149 aa loop in *E. coli* Mre11, the latter, defined as the ‘fastener’ [83], not conserved in eukaryotes (Fig. S2B and Fig. 7A, see below). This interface is claimed to be essential for both endonuclease and exonuclease activity of the complex, since mutants losing this interaction, such as Rad50^E115K^ or Mre11^K149E^, are defective in both [83]. Actually, this model was demonstrated to be suitable for binding only to DSB ends [83].

It is still to be assessed if this configuration is also topologically compatible with DNA binding far from the DSB end, which is a prerequisite for Mre11 nucleolytic activity, since Mre11 endonuclease cuts from 40 up to 200–300 nucleotides from the DSB end [20], [21], [22], [85], [86], [87]. Furthermore, the abundant Ku heterodimer is generally believed to be the first protein to bind to DSBs at least in mammalian cells [88], [89], [90], [91], [92], allowing NHEJ to make the first DSB repair attempt [91]. Experimental data in mammalian cells suggest that the MRN complex can attach to DNA ends that have already been claimed by Ku, which forms a constitutive ring specific for DNA ends and unfit for internal DNA binding [93]. In budding yeast, the absence of Ku weakens the requirement for MRX in DNA end resection to process “clean” DNA ends, suggesting that the two complexes compete for binding to DSB ends [94], [95], [96], [97], [98]. The structural traits allowing eukaryotic MR to interact with DNA ends covered by Ku or other associated proteins remain yet to be defined.

## 4 Interaction of Rad50 and Mre11 with DNA

The MR complex was previously proved to bind to dsDNA efficiently only in presence of ATP [50]. This is consistent with Rad50 interacting primarily with dsDNA in the ATP-dependent dimer conformation [99]. In this closed conformation, Rad50 dimer creates a groove that can host dsDNA, binding it with a patch of several positive residues both on the globular head and on the proximal CC surface (Fig. 6) [50], [52], [54], [83], [84].

Nonetheless, the MR complex was previously reported to make contact with DNA even in absence of any nucleotide, as observed by electron microscopy or AFM [39], [82]. The large majority of the coiled-coils in DNA-associated MR complex were rod-shaped either in the presence or in the absence of a non-hydrolysable analogue of ATP [82]. Most recently, the same rod-shaped conformation was proposed for the DNA end-bound post-ATP hydrolysis cutting state of MR complex (Fig. 4D and E) [83]. Although it is not clear if the rod-shaped conformation of the coiled-coils has any requirements as for the nucleotide binding in Rad50 dimer, it is clear that it involves a tight contact with nucleosome-free DNA and, likely, intact Zn^2+^-hooked CC. In fact, crystals obtained with shorter versions of Rad50 proteins in presence of dsDNA do not reveal significant contacts between the proximal CC domains and dsDNA (Fig. 6A) [52], [54]. Rad50 CC was found to have flexibility properties that could explain its structural dynamic nature [40]. Since the coiled-coils are generally not stiff [37], [38], they hardly can transmit changes in conformation of the globular domain to changes in relative orientation of the ends of the coiled-coil, as proposed [39], [48]. This would confirm that DNA interaction is a key force required for circular coiled-coils transition to rod-like configuration.

**Fig. 8:**
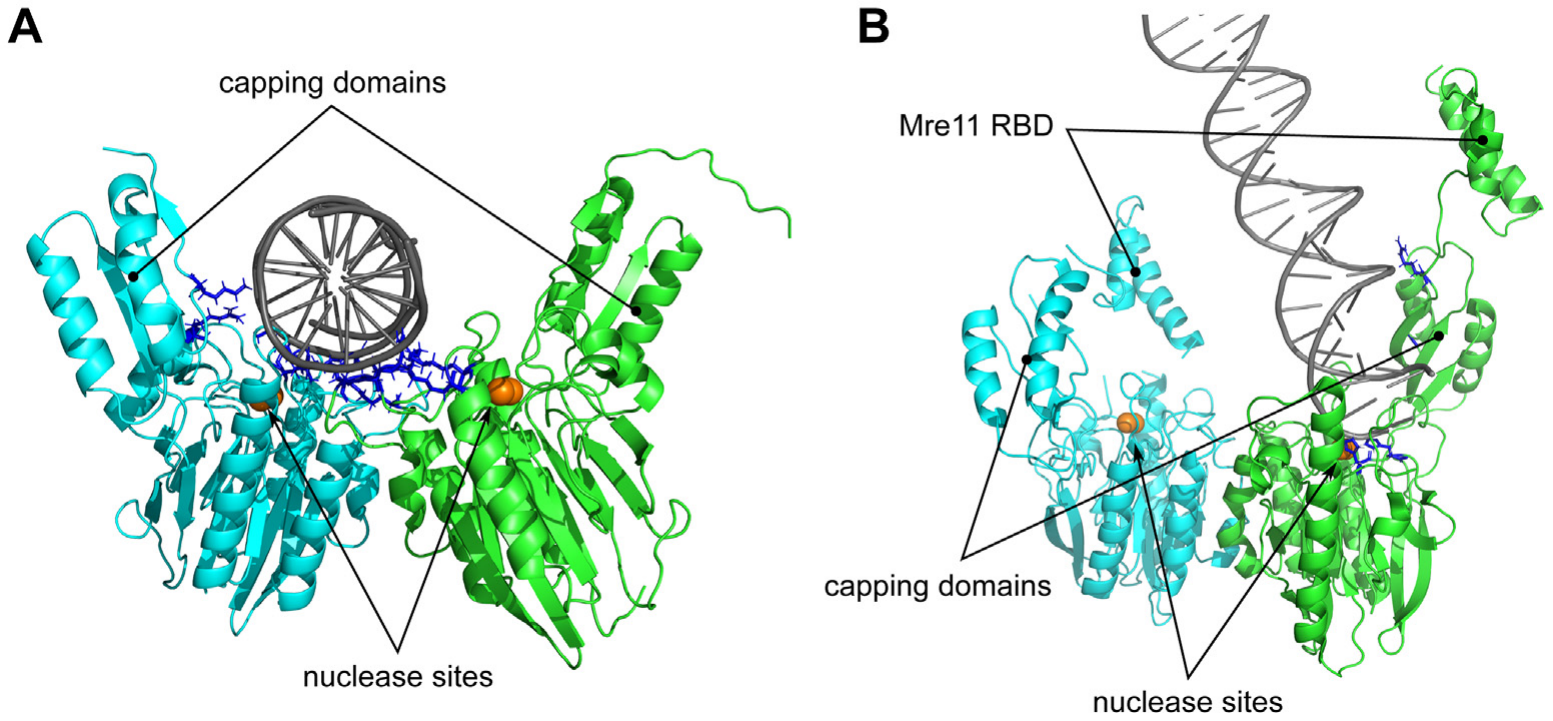
Structural data agree on Mre11 capping domain contacting dsDNA. (A) Structure of *M. jannaschii* Mre11 dimer in complex with dsDNA (PDB ID: 4TUG). (B) Detail of Mre11 interface with dsDNA end in *E. coli* M_2_R_2_ complex in cutting state configuration (PDB ID: 6S85). Mre11 subunits are reported in green and cyan; dsDNA in grey. Basic amino acids (Lys, Arg and His) that can be implicated in DNA binding are shown as blue sticks. (For interpretation of the references to colour in this figure legend, the reader is referred to the web version of this article.)

DNA access to Mre11 nuclease active sites has long been a major puzzling point, since the appearance of the first tetramer closed configuration structure [53], where Rad50 subunits clearly obstructed Mre11 catalytic sites (Fig. 2A). The Mre11 capping domains were also claimed to be involved in DNA binding, according to structural data obtained with isolated Mre11 dimer together with DNA (Fig. 8A) [13], which was recently confirmed by genetic and molecular dynamics simulations of a Mre11 mutant able to overcome some of *sae2*Δ mutant defects [100]. The clearance of the Mre11 nuclease catalytic sites is apparent in the open configuration structure described by Lammens et al. [50] (Fig. 2D and E), and this would easily allow interaction with dsDNA according to the reported structure for DNA-bound Mre11 dimer (Fig. 8A). The most recent cutting state model described by Käshammer et al. [83] (Fig. 4D and E) describes a different configuration for dsDNA end contact with Mre11 catalytic sites, which is actually still in agreement with formerly identified contact sites (Fig. 8B).

Interestingly, high-throughput single-molecule microscopy data showed that MRN, as well as MR, complex tracks the DNA helix for free DNA ends by one-dimensional facilitated diffusion, even on nucleosome-coated DNA [101]. Rad50 would bind homoduplex DNA and promote facilitated diffusion, whereas Mre11 was found to be required for DNA end recognition and nuclease activities [101]. Thus, the ability to bind and track the DNA helix, whether on naked DNA or on DNA associated to nucleosomes, would rely on Rad50 contacts with dsDNA, while the ability to recognize clean DNA ends would be reliant on Mre11 directly taking contact with the DSB ends.

## 5 Regulation of Mre11 nuclease activity

Sae2/CtIP promotes the Mre11 endonuclease activity within the MRX complex to initiate DSB resection [23] and this function requires Sae2/CtIP phosphorylation by cyclin-dependent kinases (CDKs) in both mitosis and meiosis [24], [25], [102], [103], [104]. This CDK-dependent regulation of Sae2 activity ensures that resection only takes place in the S/G2 phase of the cell cycle when a sister chromatid is available as repair template [105], [106]. Furthermore, it also explains why NHEJ repair predominates in the G1 phase, when CDK activity and therefore DSB resection is low.

In *S. cerevisae*, CDK-mediated Sae2 phosphorylation promotes MRX nuclease by at least two distinct mechanisms. During the G1 phase of the cell cycle, Sae2 exists in an unphosphorylated state and is part of an inactive soluble multimeric complex [107]. During S phase in mitosis and prophase of the first meiotic division, Sae2 phosphorylation promotes the formation of Sae2 tetramers, which likely represent the active Sae2 species that promote the Mre11 nuclease within the MRX complex [25], [107], [108], [109], [110]. Additionally and independently of regulating Sae2 size distribution, phosphorylation of the conserved C-terminal domain of Sae2 is necessary for a direct physical interaction with Rad50 [25], which is crucial to promote the Mre11 endonuclease activity. As ATP hydrolysis by Rad50 is necessary for MRX-Sae2 endonuclease [23], phosphorylated Sae2 may control the Mre11 nuclease by promoting productive ATP hydrolysis.

In vitro, the efficiency of Sae2/CtIP-induced Mre11 endonucleolytic activity is strongly enhanced by the presence of proteins stably bound either internally or at the end of a DNA molecule [86], [87], [111]. The *E. coli* SbcCD nuclease can cleave dsDNA past protein blocks as well, indicating that this seems to be a conserved mechanism [112]. Such protein blocks include histones, the Ku complex bound at the DSB ends, the RPA complex bound to either partially resected DNA ends or terminal hairpin structures, the type II topoisomerase-like Spo11 covalently bound at meiotic DSBs and the MRX/MRN complex itself [22], [23], [86], [87], [111], [113], [114], [115], [116], [117].

During meiosis, formation and repair of programmed DSBs ensures correct alignment and segregation of chromosome homologs in addition to generating diversity [1]. The meiosis-specific Spo11 protein generates DSBs by forming a covalent linkage between a conserved tyrosine residue and the 5′ end of the cleaved strand [118], [119]. In budding yeast, the MRX complex plays at least two roles during meiotic recombination. First, it is required for Spo11 to generate DSBs. Then, the Mre11 endonuclease activity and Sae2 removes Spo11 from break ends by endonucleolytic cleavage, releasing Spo11 attached to a short oligonucleotide [22], [85]. In fact, both the lack of Sae2 or of Mre11 nuclease-defective variants allow Spo11-induced DSB formation, but prevent Spo11 removal and meiotic DSB end resection in both *S. cerevisiae* and *S. pombe* cells [15], [18], [120], [121].

Interestingly, similar to *SAE2* deletion and Mre11 nuclease-defective variants, a group of *rad50* and *mre11* mutants, called *S* mutants, are proficient in meiotic DSB formation but are unable to remove Spo11 from the DSB ends (Table S1) [15], [18], [120], [121], [122], [123], [124], due to the corresponding mutations impairing MRX- Sae2 interaction not only functionally but also physically [11], [25]. Surprisingly, comparison of the structures of *S. cerevisiae* Rad50 and Mre11 (Fig. 7B), generated by homology modeling [58], with *E. coli* cutting state model (Fig. 4D and 7A) shows that these sites are localized exactly at the regions of *S. cerevisiae* Mre11 and Rad50 corresponding to the interface identified between Rad50 β sheet and Mre11 fastener loop in the DNA end binding configuration of *E. coli* MR complex. The fastener loop is hardly conserved in the other eubacterial Mre11 whose structure is available, which is *T. maritima* (PDB ID: 4NZV), while it is not present in archaea or in eukaryotes (Fig. S2B). Indeed, it is not conserved in *S. cerevisiae* as well. However, it is interesting that the T188 residue affected by the *mre11S* mutation in budding yeast [122] is localized in the Mre11 α helix facing the same region of the fastener loop (Fig. 7B). Moreover, in a *sgs1*Δ background, the R184A mutant, affecting a residue on the exposed side of the same helix (Fig. 7B), is sensitive to DNA damaging agents as a catalytic dead Mre11 mutant variant (Mre11-H125N) [125]. Thus, it was speculated that a similar configuration could be valid for eukaryotic MR complexes as well, but it could involve the formation of a ternary complex with CtIP/Sae2 [83].

It is not clear yet which structural adjustments allow Mre11 to exert its function as an endo- or an exonuclease. Separation-of-function mutants were isolated, such as H52S in *Pf* Mre11, in one of the conserved Mn^2+^-coordinating motifs, in particular in the phosphoesterase motif II (Fig. S2B), that drove to selective loss of exonuclease activity but retained endonuclease competence [126]. This conserved motif II histidine was proposed to be required for the proper rotation of the 3′-end last nucleotide phosphate bond over the catalytic Mn^2+^ ions prior to its hydrolysis. The H52S mutation was also suggested to affect an allosteric network between the Mre11 active site and capping domain, which is involved in ssDNA binding [13], [126].

Another mutant, the Y187C mutant of *Pf*Mre11, was also found to be inactive as an exonuclease but active as an endonuclease [39]. The mutation affects the conserved aromatic residue Tyr187 in *Pf*Mre11 (*S. cerevisiae* F224, *H. sapiens* F227), situated in a loop corresponding to the sealing loop important for *E. coli* MR nuclease activity [83], on the opposite side of the catalytic Mn^2+^ ions. Tyr187 was found by X-ray crystallographic structural studies to interact with the nucleotide of the dAMP released by the cleavage, while the monophosphate interacted with the Mn^2+^ ions in the catalytic site [39]. Structural studies via methyl-based solution-state NMR spectroscopy revealed that both Y187C and H52S mutations in *Pf*Mre11 alter the structural and dynamical interactions with dsDNA [126].

A series of mirin-based inhibitors binding to the same motif II were also designed that could specifically interfere with either one of the nuclease activity [19]. It was hypothesized that the small-molecule inhibitors that differentially prevented endonuclease but not exonuclease activity were able to specifically limit ssDNA binding [19].

## 6 Regulation of Exo1 recruitment and processivity

MRX/MRN-Sae2/CtIP creates a nick that provides an internal entry site for nucleases capable of degrading DNA in a 5′–3′ direction. These nucleases comprise Exo1 and Dna2, which control two partially overlapping pathways [20], [21]. While Exo1 is able to release mononucleotide products from a dsDNA end [127], Dna2 has endonuclease activity that can cleave both 5′ and 3′ single-stranded DNA overhangs adjoining a duplex region [128]. Reconstitution experiments revealed that Exo1 nuclease requires the support of various factors that promote its nuclease activity. The mismatch recognition complex MutSα was shown to stimulate Exo1 processivity in the presence of a mismatch [129], whereas the proliferating cell nuclear antigen (PCNA) promotes human EXO1 processivity by enhancing its association with DNA [30], [130]. Noteworthy, in addition to the end-clipping function, MRX/MRN also stimulates resection by Exo1/EXO1 [28], [29], [30], [31], [32], [98], thus explaining why *mre11*Δ cells show a resection defect more severe than *sae2*Δ or *mre11* nuclease defective mutants.

Human EXO1 is a processive enzyme per se, unless the generated ssDNA is rapidly associated to RPA, as usually happens within the cell, which reduces EXO1 life-time on DNA by ~100-fold [101]. In this condition, physical interaction with MRN [28], [30], [31], primarily with MRE11 and less strongly with NBS1, is required to maintain EXO1 processivity, retaining the exonuclease on DNA and allowing fully efficient long-range resection [101].

The MRX/MRN complex was early reported to exert an ATP hydrolysis-requiring partial and not processive unwinding of a short DNA duplex [17], [131], [132], [133], [134]. This limited activity seems to be conserved in prokaryotes as well [54]. Later, it was proposed that Mre11 capping domain could retain a DNA unwrapping activity, ensuring duplex melting at the DNA end, linked to capping domain rotation [13]. This movement was actually reproduced by molecular dynamics simulations of *S. cerevisiae* Mre11, and was reported to be able to cause DNA terminus unwinding [135]. The movement was exacerbated by the presence of a R10T single amino acid substitution in Mre11, which implied an altered orientation of the Mre11 capping domain leading to more persistent melting of the dsDNA end. This hyperactivation allowed a higher exonuclease activity by Exo1, achieving the suppression of the DNA damage hypersensitivity and the resection defect of *sae2*Δ cells [100]. In fact, although Exo1 is able to degrade a filament in dsDNA *in vitro*, indicating that it does not require the intervention of a helicase, it actually prefers dsDNA bearing a 3′ ssDNA overhang [32], and the DNA end unwinding by MR complex could facilitate its access to the DNA 5′ terminus.

## 7 DNA tethering

Besides DNA end processing reaction, a second key requirement of MRX/MRN complexes in DSB repair is the ability to coordinate and bridge DNA ends, achieved through the MRN complex architectural DNA scaffolding activities [39], [42], [43], [44], [45], [46], [47], [55], [82], [136]. A role in DNA bridging was also proposed for CtIP/Ctp1/Sae2 [137], [138], although it cannot substitute for MRX/MRN deficiency, and it was proposed to supersede after DSB processing.

**Fig. 9:**
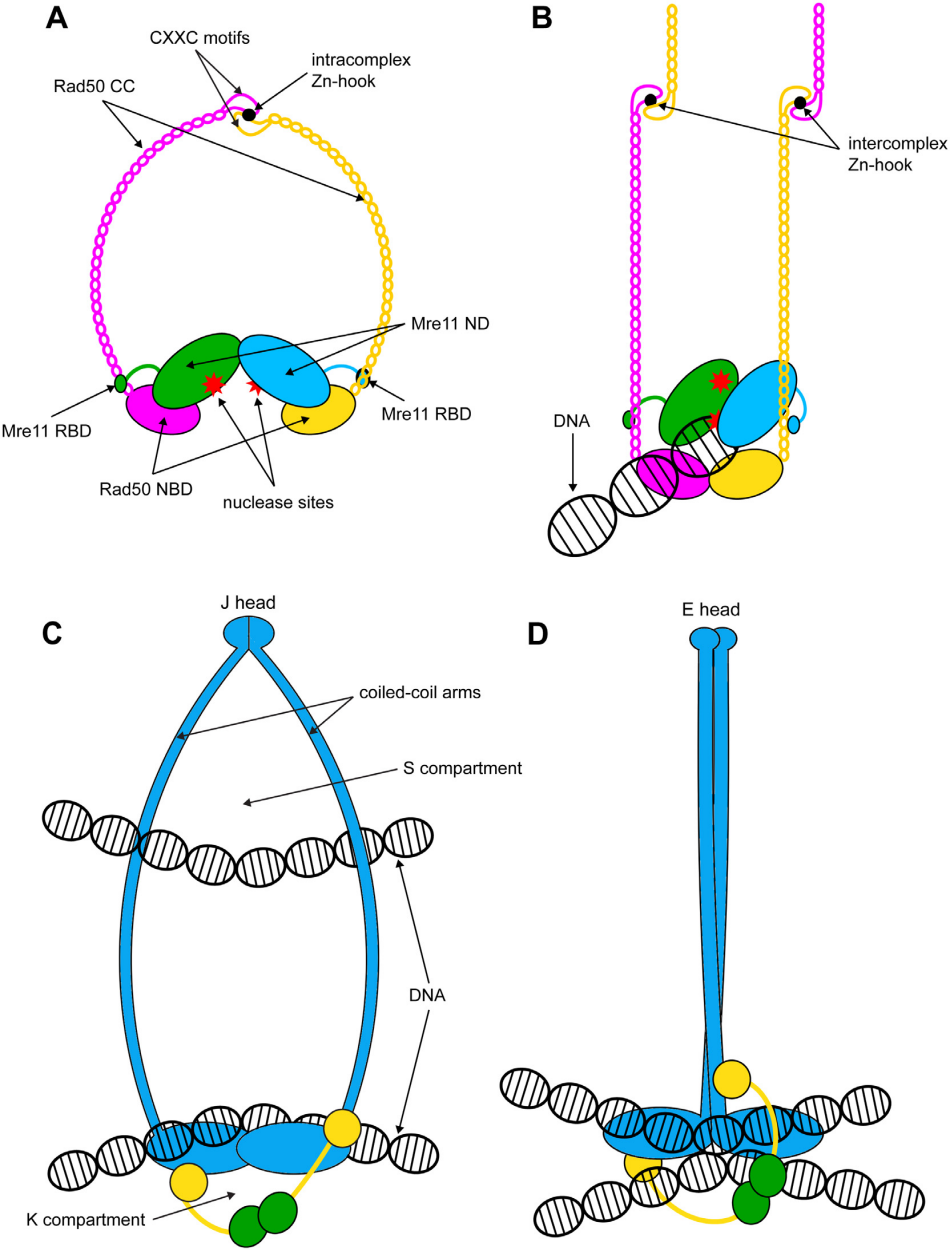
Comparison among DNA tethering models for MRX/MRN complex and SMC proteins. (A) Open configuration for MR complex. (B) Clamping model of dsDNA binding and DNA tethering by intercomplex Zn-hook dimerization. (C) Schematic representation of the DNA tethering structure observed for the *Bacillus subtilis* SMC (SMC-ScpAB, SMC is in blue and the kleisin family proteins ScpA and B are in yellow and green) complex with J head conformation and overall V shape configuration. (D) Schematic representation of the DNA tethering structure observed for SMC-ScpAB (same colors as in panel C) with E head configuration, leading to the so called I shape configuration. (For interpretation of the references to colour in this figure legend, the reader is referred to the web version of this article.)

The DNA tethering function for the MRX/MRN complex is generally ascribed to the extended coiled-coil regions of Rad50 [37], [39], [45], [136] and requires the Rad50 hook domain [39], [44], [139]. These observations lead to a model describing in trans bridging of DNA molecules through an alternative arrangement of the Zn-hook interface. In the so-called intercomplex configuration (Fig. S3), the coiled coils of the Rad50 subunits in one heterotetrameric complex connect at the hook with the coiled coils of the Rad50 subunits from another complex, effectively bridging two molecules of DNA (compare Fig. 9A and B).

Other data revealed that the hook alone would not be sufficient for establishing of DNA tethering, which rather involves Rad50 coiled-coils as well. For instance, although mutations in the cysteine residues in the hook actually impair MR complex assembly and chromatin binding in mammals [47], [61], this is not sufficient in yeast [44], [140], where simultaneous loss of hook and coiled-coil distal interface is required to induce a phenotype similar to *rad50* loss-of-function both in sister chromatid recombination and in NHEJ [48]. Previously, dramatic shortening of the coiled-coils in yeast, though in presence of the hook domain and thus compatible with intercomplex hook-mediated dimerization, was reported to be sufficient to dramatically impair NHEJ, which requires MRX/MRN to tether the two DNA ends together, but not to process them [44].

Moreover, observation by AFM of nucleoproteins formed by incubation of 400 bp-dsDNA with MRX particles revealed that internal and terminal nucleoprotein complexes had similar size, suggesting that the DNA molecules were held together by a single MRX complex [141]. And finally, very recently, high-speed AFM imaging of the human MR complex has shown that the Rad50 coiled-coil arms are stably bridged by the dimerized hooks, while the MR ring rather opens by disconnecting the head domains [142], [143].

Taken together, these considerations raise an issue on the possibility that DNA tethering could be achieved in different manners depending on the DNA repair pathway to be undertaken. DNA binding by clamping, as proposed by Lammens et al. [50] (Fig. 9B), and in general dsDNA making contact with Rad50 proximal coiled-coils and DNA binding cleft on its dimer heads presume that DNA is actually protein-free, which would be achieved near the DNA ends by chromatin remodeling and histones eviction [144], [145], [146]. Recently, CC clamping on the DNA helix was actually observed by cryo-EM, but only after ATP hydrolysis [83]. Hitherto, no structural evidence allows to predict if DNA molecule actually makes contact with Rad50 proximal CC in the ATP-bound state, although the presence of ATP is necessary to enforce MRN binding to DNA [49], [132].

Indeed, the ATP-driven Rad50 dimerization promotes the assembly of MRX/MRN globular domain and has previously been proposed to allow to topologically encircle homoduplex DNA within the ring-shaped CC compartment [52], [54], similar to the model envisioned for S compartments defined by cohesin heads in J configuration [147], [148], [149], [150] (Fig. 9C). Like its homolog Rad50, cohesin displays two ~30- to 50-nm-long coiled coils as well. Nonetheless, human and *S. pombe* cohesins were reported to fail to overcome diverse roadblocks [151], [152]. In contrast, MRX/MRN, unless Rad50 coiled-coils extension is reduced [101], can efficiently diffuse on nucleosome arrays, provided that they are not too dense, which ensures that MRX/MRN can rapidly find DSB ends in euchromatin and nucleosome-depleted genomic regions. Reduced MRN diffusion on highly chromatinized DNA may indeed contribute to scarce HR and delayed repair at heterochromatic DNA breaks (see [5] for a recent review on chromatin context in DSB processing).

Previous cross-linking studies confirmed this topology for DNA capture in the S compartment inside SMC rings in eukaryotic cohesin [153] or prokaryotic condensin [154] in a V shape conformation (Fig. 9C). However, after ATP hydrolysis the yeast cohesin heads adopt a E configuration and the coiled coils seem to be juxtaposed throughout their length, adopting the I shape conformation, for bacterial Smc proteins and eukaryotic cohesin as well [155], [156], [157]. The I shape configuration was claimed to hamper DNA entrapment in S compartment and allowing DNA to enter the K compartment, defined by Smc and kleisin heads [142] (Fig. 9D). Consequently, it has been recently proposed that the long, flexible arms of SMC-like proteins would be fundamental to allow DNA translocation by mediating large steps on chromatin, rather than to embrace chromosomal DNA fibers [154], which is a fascinating hypothesis for Rad50 proteins as well. Further experiments will be required to assess if this bimodal DNA tethering mechanism could be adopted by MR complexes.

## 8 Summary and outlook

DNA damage and DNA repair are fundamental topics in genetics and molecular biology due to their correlation with genomic instability and DNA mutations. Cells possess mechanisms conserved among eukaryotes apt to recognize DSBs and promote their repair. Misrepair of DSBs often leads genomic instability and loss of genetic information that can result in cell death or oncogenic transformation. Consistently, cancer cells have a higher DSB generation rate related to oncogene-induced replication stress and dramatically rely on efficient DSB repair for their survival [158]. To date, a number of inhibitors related to DNA damage repair systems have been developed, particularly for breast cancer [159]. Understanding the mechanism involved in DNA damage repair would be extremely useful in order to identify novel targets for drug discovery efforts.

Events driving DSB-triggered MR-driven ATP hydrolysis and subsequent DNA access to Mre11 endonuclease site have not been clarified yet, despite the joined effort of genetics, biochemical, biophysical, computational, and structural approaches. In particular, the conformational transition recently proposed for *E. coli* MR complex upon recognition of the DNA end poses a novel focus on the molecular rearrangements that can either trigger or allow such transition. Extending this framework to eukaryotic MR complex will be a further and challenging step required to transfer and compare available experimental data on the different molecular systems.

Computational approaches such as molecular dynamics simulations contributed to this issue by providing unique insights on the MR components molecular properties. Indeed, further structural insights in eukaryotic complexes, which display specific traits totally absent in archaea and prokaryotic homologs, will be required to experimentally validate predictions obtained in these homologs by advanced structural analysis techniques. Future investigation will enable to determine whether the novel conformational rearrangement proposed for bacterial MR complex could be envisioned for eukaryotic MR complexes as well, allowing to exploit the available structural insight and transfer the novel concept to other organisms systems.

## Declaration of Competing Interest

The authors declare that they have no known competing financial interests or personal relationships that could have appeared to influence the work reported in this paper.

## References

[1] Lam I., Keeney S. (2015). Mechanism and regulation of meiotic recombination initiation. Cold Spring Harb Perspect Biol.

[2] Arya R., Bassing C.H. (2017). V(D)J recombination exploits DNA damage responses to promote immunity. Trends Genet.

[3] Chang H.H.Y., Pannunzio N.R., Adachi N., Lieber M.R. (2017). Non-homologous DNA end joining and alternative pathways to double-strand break repair. Nat Rev Mol Cell Biol.

[4] Kowalczykowski S.C. (2015). An overview of the molecular mechanisms of recombinational DNA repair. Cold Spring Harb Perspect Biol.

[5] Casari E., Rinaldi C., Marsella A., Gnugnoli M., Colombo C.V., Bonetti D. (2019). Processing of DNA double-strand breaks by the MRX complex in a chromatin context. Front Mol Biosci.

[6] Syed A., Tainer J.A. (2018). The MRE11-RAD50-NBS1 complex conducts the orchestration of damage signaling and outcomes to stress in DNA replication and repair. Annu Rev Biochem.

[7] Villa M., Cassani C., Gobbini E., Bonetti D., Longhese M.P. (2016). Coupling end resection with the checkpoint response at DNA double-strand breaks. Cell Mol Life Sci.

[8] Ritchie K.B., Petes T.D. (2000). The Mre11p/Rad50p/Xrs2p complex and the Tellp function in a single pathway for telomere maintenance in yeast. Genetics.

[9] Kolinjivadi A.M., Sannino V., de Antoni A., Técher H., Baldi G., Costanzo V. (2017). Moonlighting at replication forks - a new life for homologous recombination proteins BRCA1, BRCA2 and RAD51. FEBS Lett.

[10] Pasero P., Vindigni A. (2017). Nucleases acting at stalled forks: how to reboot the replication program with a few shortcuts. Annu Rev Genet.

[11] Hopfner K.P., Karcher A., Craig L., Woo T.T., Carney J.P., Tainer J.A. (2001). Structural biochemistry and interaction architecture of the DNA double-strand break repair Mre11 nuclease and Rad50-ATPase. Cell.

[12] Moncalian G., Lengsfeld B., Bhaskara V., Hopfner K.P., Karcher A., Alden E. (2004). The Rad50 signature motif: essential to ATP binding and biological function. J Mol Biol.

[13] Williams R.S., Moncalian G., Williams J.S., Yamada Y., Limbo O., Shin D.S. (2008). Mre11 dimers coordinate DNA end bridging and nuclease processing in double-strand-break repair. Cell.

[14] Bressan D.A., Olivares H.A., Nelms B.E., Petrini J.H. (1998). Alteration of N-terminal phosphoesterase signature motifs inactivates *Saccharomyces cerevisiae* Mre11. Genetics.

[15] Furuse M., Nagase Y., Tsubouchi H., Murakami-Murofushi K., Shibata T., Ohta K. (1998). Distinct roles of two separable in vitro activities of yeast Mre11 in mitotic and meiotic recombination. EMBO J.

[16] Paull T.T., Gellert M. (1998). The 3′ to 5′ exonuclease activity of Mre11 facilitates repair of DNA double-strand breaks. Mol Cell.

[17] Trujillo K.M., Yuan S.S.F., Lee E.Y.H.P., Sung P. (1998). Nuclease activities in a complex of human recombination and DNA repair factors Rad50, Mre11, and p95. J Biol Chem.

[18] Usui T., Ohta T., Oshiumi H., Tomizawa J.I., Ogawa H., Ogawa T. (1998). Complex formation and functional versatility of Mre11 of budding yeast in recombination. Cell.

[19] Shibata A., Moiani D., Arvai A.S., Perry J., Harding S.M., Genois M.M. (2014). DNA double-strand break repair pathway choice is directed by distinct MRE11 nuclease activities. Mol Cell.

[20] Mimitou E.P., Symington L.S. (2008). Sae2, Exo1 and Sgs1 collaborate in DNA double-strand break processing. Nature.

[21] Zhu Z., Chung W.H., Shim E.Y., Lee S.E., Ira G. (2008). Sgs1 helicase and two nucleases Dna2 and Exo1 resect DNA double-Strand break ends. Cell.

[22] Garcia V., Phelps S.E.L., Gray S., Neale M.J. (2011). Bidirectional resection of DNA double-strand breaks by Mre11 and Exo1. Nature.

[23] Cannavo E., Cejka P. (2014). Sae2 promotes dsDNA endonuclease activity within Mre11-Rad50-Xrs2 to resect DNA breaks. Nature.

[24] Anand R., Ranjha L., Cannavo E., Cejka P. (2016). Phosphorylated CtIP functions as a co-factor of the MRE11-RAD50-NBS1 endonuclease in DNA end resection. Mol Cell.

[25] Cannavo E., Johnson D., Andres S.N., Kissling V.M., Reinert J.K., Garcia V. (2018). Regulatory control of DNA end resection by Sae2 phosphorylation. Nat Commun.

[26] Sturzenegger A., Burdova K., Kanagaraj R., Levikova M., Pinto C., Cejka P. (2014). DNA2 cooperates with the WRN and BLM RecQ helicases to mediate long-range DNA end resection in human cells. J Biol Chem.

[27] Gravel S., Chapman J.R., Magill C., Jackson S.P. (2008). DNA helicases Sgs1 and BLM promote DNA double-strand break resection. Genes Dev.

[28] Cejka P., Cannavo E., Polaczek P., Masuda-Sasa T., Pokharel S., Campbell J.L. (2010). DNA end resection by Dna2-Sgs1-RPA and its stimulation by Top3-Rmi1 and Mre11-Rad50-Xrs2. Nature.

[29] Nicolette M.L., Lee K., Guo Z., Rani M., Chow J.M., Lee S.E. (2010). Mre11-Rad50-Xrs2 and Sae2 promote 5′ strand resection of DNA double-strand breaks. Nat Struct Mol Biol.

[30] Nimonkar A.V., Genschel J., Kinoshita E., Polaczek P., Campbell J.L., Wyman C. (2011). BLM-DNA2-RPA-MRN and EXO1-BLM-RPA-MRN constitute two DNA end resection machineries for human DNA break repair. Genes Dev.

[31] Niu H., Chung W.H., Zhu Z., Kwon Y., Zhao W., Chi P. (2010). Mechanism of the ATP-dependent DNA end resection machinery from *Saccharomyces cerevisiae*. Nature.

[32] Cannavo E., Cejka P., Kowalczykowski S.C. (2013). Relationship of DNA degradation by *Saccharomyces cerevisiae* exonuclease 1 and its stimulation by RPA and Mre11-Rad50-Xrs2 to DNA end resection. Proc Natl Acad Sci USA.

[33] O’Driscoll M. Diseases associated with defective responses to DNA damage. Cold Spring Harb Perspect Biol 2012;4. https://doi.org/10.1101/cshperspect.a012773. 10.1101/cshperspect.a012773 PMC3504433 23209155

[34] Stracker T.H., Petrini J.H.J. (2011). The MRE11 complex: starting from the ends. Nat Rev Mol Cell Biol.

[35] Paull T.T. (2018). 20 years of Mre11 biology: no end in sight. Mol Cell.

[36] Anderson D.E., Trujillo K.M., Sung P., Erickson H.P. (2001). Structure of the Rad50·Mre11 DNA repair complex from *Saccharomyces cerevisiae* by electron microscopy. J Biol Chem.

[37] de Jager M., Van Noort J., Van Gent D.C., Dekker C., Kanaar R., Wyman C. (2001). Human Rad50/Mre11 is a flexible complex that can tether DNA ends. Mol Cell.

[38] de Jager M., Trujillo K.M., Sung P., Hopfner K.P., Carney J.P., Tainer J.A. (2004). Differential arrangements of conserved building blocks among homologs of the Rad50/Mre11 DNA repair protein complex. J Mol Biol.

[39] Hopfner K.P., Craig L., Moncalian G., Zinkel R.A., Usui T., Owen B.A. (2002). The Rad50 zinc-hook is a structure joining Mre11 complexes in DNA recombination and repair. Nature.

[40] Van Noort J., Van der Heijden T., De Jager M., Wyman C., Kanaar R., Dekker C. (2003). The coiled-coil of the human Rad50 DNA repair protein contains specific segments of increased flexibility. Proc Natl Acad Sci USA.

[41] Williams R.S., Tainer J.A. (2005). A nanomachine for making ends meet: MRN is a flexing scaffold for the repair of DNA double-strand breaks. Mol Cell.

[42] Kaye J.A., Melo J.A., Cheung S.K., Vaze M.B., Haber J.E., Toczyski D.P. (2004). DNA breaks promote genomic instability by impeding proper chromosome segregation. Curr Biol.

[43] Lobachev K., Vitriol E., Stemple J., Resnick M.A., Bloom K. (2004). Chromosome fragmentation after induction of a double-strand break is an active process prevented by the RMX repair complex. Curr Biol.

[44] Wiltzius J.J.W., Hohl M., Fleming J.C., Petrini J.H.J. (2005). The Rad50 hook domain is a critical determinant of Mre11 complex functions. Nat Struct Mol Biol.

[45] Hohl M., Kwon Y., Galván S.M., Xue X., Tous C., Aguilera A. (2010). The Rad50 coiled-coil domain is indispensable for Mre11 complex functions. Nat Struct Mol Biol.

[46] Nakai W., Westmoreland J., Yeh E., Bloom K., Resnick M.A. (2011). Chromosome integrity at a double-strand break requires exonuclease 1 and MRX. DNA Repair (Amst).

[47] He J., Shi L.Z., Truong L.N., Lu C.S., Razavian N., Li Y. (2012). Rad50 zinc hook is important for the Mre11 complex to bind chromosomal DNA double-stranded breaks and initiate various DNA damage responses. J Biol Chem.

[48] Park Y.B., Hohl M., Padjasek M., Jeong E., Jin K.S., Krȩzel A. (2017). Eukaryotic Rad50 functions as a rod-shaped dimer. Nat Struct Mol Biol.

[49] Hopfner K.P., Karcher A., Shin D.S., Craig L., Arthur L.M., Carney J.P. (2000). Structural biology of Rad50 ATPase: ATP-driven conformational control in DNA double-strand break repair and the ABC-ATPase superfamily. Cell.

[50] Lammens K., Bemeleit D.J., Möckel C., Clausing E., Schele A., Hartung S. (2011). The Mre11:Rad50 structure shows an ATP-dependent molecular clamp in DNA double-strand break repair. Cell.

[51] Williams G.J., Williams R.S., Williams J.S., Moncalian G., Arvai A.S., Limbo O. (2011). ABC ATPase signature helices in Rad50 link nucleotide state to Mre11 interface for DNA repair. Nat Struct Mol Biol.

[52] Seifert F.U., Lammens K., Stoehr G., Kessler B., Hopfner K. (2016). Structural mechanism of ATP-dependent DNA binding and DNA end bridging by eukaryotic Rad50. EMBO J.

[53] Lim H.S., Kim J.S., Park Y.B., Gwon G.H., Cho Y. (2011). Crystal structure of the Mre11-Rad50-ATPγS complex: understanding the interplay between Mre11 and Rad50. Genes Dev.

[54] Liu Y., Sung S., Kim Y., Li F., Gwon G., Jo A. (2016). ATP-dependent DNA binding, unwinding, and resection by the Mre11/Rad50 complex. EMBO J.

[55] Deshpande R.A., Williams G.J., Limbo O., Williams R.S., Kuhnlein J., Lee J.-H. (2014). ATP-driven Rad50 conformations regulate DNA tethering, end resection, and ATM checkpoint signaling. EMBO J.

[56] Möckel C., Lammens K., Schele A., Hopfner K.-P. (2012). ATP driven structural changes of the bacterial Mre11:Rad50 catalytic head complex. Nucleic Acids Res.

[57] Krogh B.O., Llorente B., Lam A., Symington L.S. (2005). Mutations in Mre11 phosphoesterase motif I that impair *Saccharomyces cerevisiae* Mre11-Rad50-Xrs2 complex stability in addition to nuclease activity. Genetics.

[58] Cassani C., Gobbini E., Vertemara J., Wang W., Marsella A., Sung P. (2018). Structurally distinct Mre11 domains mediate MRX functions in resection, end-tethering and DNA damage resistance. Nucleic Acids Res.

[59] Barfoot T. (2015). Functional analysis of the bacteriophage T4 Rad50 homolog (gp46) coiled-coil domain. J Biol Chem.

[60] Hohl M. (2015). Interdependence of the rad50 hook and globular domain functions. Mol Cell.

[61] Roset R., Inagaki A., Hohl M., Brenet F., Lafrance-Vanasse J., Lange J. (2014). The Rad50 hook domain regulates DNA damage signaling and tumorigenesis. Genes Dev.

[62] Carney J.P., Maser R.S., Olivares H., Davis E.M., Le Beau M., Yates J.R. (1998). The hMre11/hRad50 protein complex and Nijmegen breakage syndrome: linkage of double-strand break repair to the cellular DNA damage response. Cell.

[63] Desai-Mehta A., Cerosaletti K.M., Concannon P. (2001). Distinct functional domains of Nibrin mediate Mre11 binding, focus formation, and nuclear localization. Mol Cell Biol.

[64] Nakada D., Matsumoto K., Sugimoto K. (2003). ATM-related Tel1 associates with double-strand breaks through an Xrs2-dependent mechanism. Genes Dev.

[65] Falck J., Coates J., Jackson S.P. (2005). Conserved modes of recruitment of ATM, ATR and DNA-PKCs to sites of DNA damage. Nature.

[66] Tsukamoto Y., Mitsuoka C., Terasawa M., Ogawa H., Ogawa T. (2005). Xrs2p regulates Mre11p translocation to the nucleus and plays a role in telomere elongation and meiotic recombination. Mol Biol Cell.

[67] You Z., Chahwan C., Bailis J., Hunter T., Russell P. (2005). ATM activation and its recruitment to damaged DNA require binding to the C terminus of Nbs1. Mol Cell Biol.

[68] Schiller C.B., Lammens K., Guerini I., Coordes B., Feldmann H., Schlauderer F. (2012). Structure of Mre11-Nbs1 complex yields insights into ataxia-telangiectasia- like disease mutations and DNA damage signaling. Nat Struct Mol Biol.

[69] Kobayashi J., Tauchi H., Sakamoto S., Nakamura A., Morishima K., Matsuura S. (2002). NBS1 localizes to gamma-H2AX foci through interaction with the FHA/BRCT domain. Curr Biol.

[70] Melander F., Bekker-Jensen S., Falck J., Bartek J., Mailand N., Lukas J. (2008). Phosphorylation of SDT repeats in the MDC1 N terminus triggers retention of NBS1 at the DNA damage-modified chromatin. J Cell Biol.

[71] Spycher C., Miller E.S., Townsend K., Pavic L., Morrice N.A., Janscak P. (2008). Constitutive phosphorylation of MDC1 physically links the MRE11-RAD50 NBS1 complex to damaged chromatin. J Cell Biol.

[72] Chapman J.R., Jackson S.P. (2008). Phospho-dependent interactions between NBS1 and MDC1 mediate chromatin retention of the MRN complex at sites of DNA damage. EMBO Rep.

[73] Williams R.S., Dodson G.E., Limbo O., Yamada Y., Williams J.S., Guenther G. (2009). Nbs1 flexibly tethers Ctp1 and Mre11-Rad50 to coordinate DNA double-strand break processing and repair. Cell.

[74] Lloyd J., Chapman J.R., Clapperton J.A., Haire L.F., Hartsuiker E., Li J. (2009). A supramodular FHA/BRCT-repeat architecture mediates Nbs1 adaptor function in response to DNA damage. Cell.

[75] Anand R., Jasrotia A., Bundschuh D., Howard S.M., Ranjha L., Stucki M. (2019). NBS1 promotes the endonuclease activity of the MRE11-RAD50 complex by sensing CtIP phosphorylation. EMBO J.

[76] Oh J., Al-Zain A., Cannavo E., Cejka P., Symington L.S. (2016). Xrs2 dependent and independent functions of the Mre11-Rad50 complex. Mol Cell.

[77] Boswell Z.K., Rahman S., Canny M.D., Latham M.P. (2018). A dynamic allosteric pathway underlies Rad50 ABC ATPase function in DNA repair. Sci Rep.

[78] Cassani C., Vertemara J., Bassani M., Marsella A., Tisi R., Zampella G. (2019). The ATP-bound conformation of the Mre11-Rad50 complex is essential for Tel1/ATM activation. Nucleic Acids Res.

[79] Boswell Z.K., Canny M.D., Buschmann T.A., Sang J., Latham M.P. (2020). Adjacent mutations in the archaeal Rad50 ABC ATPase D-loop disrupt allosteric regulation of ATP hydrolysis through different mechanisms. Nucleic Acids Res.

[80] Al-Ahmadie H., Iyer G., Hohl M., Asthana S., Inagaki A., Schultz N. (2014). Synthetic lethality in ATM-deficient RAD50-mutant tumors underlies outlier response to cancer therapy. Cancer Discov.

[81] Lee J.H., Mand M.R., Deshpande R.A., Kinoshita E., Yang S.H., Wyman C. (2013). Ataxia Telangiectasia-Mutated (ATM) kinase activity is regulated by ATP-driven conformational changes in the Mre11/Rad50/Nbs1 (MRN) complex. J Biol Chem.

[82] Moreno-Herrero F., De Jager M., Dekker N.H., Kanaar R., Wyman C., Dekker C. (2005). Mesoscale conformational changes in the DNA-repair complex Rad50/Mre11/Nbs1 upon binding DNA. Nature.

[83] Käshammer L., Saathoff J.H., Lammens K., Gut F., Bartho J., Alt A. (2019). Mechanism of DNA end sensing and processing by the Mre11-Rad50 Complex. Mol Cell.

[84] Rojowska A., Lammens K., Seifert F.U., Direnberger C., Feldmann H., Hopfner K. (2014). Structure of the Rad50 DNA double-strand break repair protein in complex with DNA. EMBO J.

[85] Neale M.J., Pan J., Keeney S. (2005). Endonucleolytic processing of covalent protein-linked DNA double-strand breaks. Nature.

[86] Wang W., Daley J.M., Kwon Y., Krasner D.S., Sung P. (2017). Plasticity of the Mre11-Rad50-Xrs2-Sae2 nuclease ensemble in the processing of DNA-bound obstacles. Genes Dev.

[87] Reginato G., Cannavo E., Cejka P. (2017). Physiological protein blocks direct the Mre11-Rad50-Xrs2 and Sae2 nuclease complex to initiate DNA end resection. Genes Dev.

[88] Lieber M.R. (2008). The mechanism of human nonhomologous DNA end joining. J Biol Chem.

[89] Roberts S.A., Ramsden D.A. (2007). Loading of the nonhomologous end joining factor, Ku, on protein-occluded DNA ends. J Biol Chem.

[90] Beucher A., Birraux J., Tchouandong L., Barton O., Shibata A., Conrad S. (2009). ATM and Artemis promote homologous recombination of radiation-induced DNA double-strand breaks in G2. EMBO J.

[91] Shibata A., Conrad S., Birraux J., Geuting V., Barton O., Ismail A. (2011). Factors determining DNA double-strand break repair pathway choice in G2 phase. EMBO J.

[92] Kochan J.A., Desclos E.C.B., Bosch R., Meister L., Vriend L.E.M., van Attikum H. (2017). Meta-analysis of DNA double-strand break response kinetics. Nucleic Acids Res.

[93] Walker J.R., Corpina R.A., Goldberg J. (2001). Structure of the Ku heterodimer bound to DNA and its implications for double-strand break repair. Nature.

[94] Bonetti D., Clerici M., Manfrini N., Lucchini G., Longhese M.P. (2010). The MRX complex plays multiple functions in resection of Yku- and Rif2-protected DNA ends. PLoS ONE.

[95] Clerici M., Mantiero D., Guerini I., Lucchini G., Longhese M.P. (2008). The Yku70–Yku80 complex contributes to regulate double-strand break processing and checkpoint activation during the cell cycle. EMBO Rep.

[96] Foster S.S., Balestrini A., Petrini J.H.J. (2011). Functional interplay of the Mre11 nuclease and Ku in the response to replication-associated DNA damage. Mol Cell Biol.

[97] Mimitou E.P., Symington L.S. (2010). Ku prevents Exo1 and Sgs1-dependent resection of DNA ends in the absence of a functional MRX complex or Sae2. EMBO J.

[98] Shim E.Y., Chung W.-H., Nicolette M.L., Zhang Y., Davis M., Zhu Z. (2010). *Saccharomyces cerevisiae* Mre11/Rad50/Xrs2 and Ku proteins regulate association of Exo1 and Dna2 with DNA breaks. EMBO J.

[99] Hopfner K.P., Karcher A., Shin D., Fairley C., Tainer J.A., Carney J.P. (2000). Mre11 and Rad50 from *Pyrococcus furiosus*: cloning and biochemical characterization reveal an evolutionarily conserved multiprotein machine. J Bacteriol.

[100] Gobbini E., Cassani C., Vertemara J., Wang W., Mambretti F., Casari E. (2018). The MRX complex regulates Exo1 resection activity by altering DNA end structure. EMBO J.

[101] Myler L.R., Gallardo I.F., Soniat M.M., Deshpande R.A., Gonzalez X.B., Kim Y. (2017). Single-molecule imaging reveals how Mre11-Rad50-Nbs1 initiates DNA break repair. Mol Cell.

[102] Cartagena-Lirola H., Guerini I., Viscardi V., Lucchini G., Longhese M.P. (2006). Budding yeast Sae2 is an in vivo target of the Mec1 and Tel1 checkpoint kinases during meiosis. Cell Cycle.

[103] Huertas P., Cortés-Ledesma F., Sartori A.A., Aguilera A., Jackson S.P. (2008). CDK targets Sae2 to control DNA-end resection and homologous recombination. Nature.

[104] Huertas P., Jackason S.P. (2009). Human CtIP mediates cell cycle control of DNA end resection and double strand break repair. J Biol Chem.

[105] Aylon Y., Liefshitz B., Kupiec M. (2004). The CDK regulates repair of double-strand breaks by homologous recombination during the cell cycle. EMBO J.

[106] Ira G., Pellicioli A., Balijja A., Wang X., Fiorani S., Carotenuto W. (2004). DNA end resection, homologous recombination and DNA damage checkpoint activation require CDK1. Nature.

[107] Fu Q., Chow J., Bernstein K.A., Makharashvili N., Arora S., Lee C.-F. (2014). Phosphorylation-regulated transitions in an oligomeric state control the activity of the Sae2 DNA repair enzyme. Mol Cell Biol.

[108] Andres S.N., Appel C.D., Westmoreland J.W., Williams J.S., Nguyen Y., Robertson P.D. (2015). Tetrameric Ctp1 coordinates DNA binding and DNA bridging in DNA double-strand-break repair. Nat Struct Mol Biol.

[109] Davies O.R., Forment J.V., Sun M., Belotserkovskaya R., Coates J., Galanty Y. (2015). CtIP tetramer assembly is required for DNA-end resection and repair. Nat Struct Mol Biol.

[110] Kim H.S., Vijayakumar S., Reger M., Harrison J.C., Haber J.E., Weil C. (2008). Functional interactions between Sae2 and the Mre11 complex. Genetics.

[111] Cannavo E., Reginato G., Cejka P. (2019). Stepwise 5′ DNA end-specific resection of DNA breaks by the Mre11-Rad50-Xrs2 and Sae2 nuclease ensemble. Proc Natl Acad Sci U S A.

[112] Connelly J., de Leau E., Leach D. (2003). Nucleolytic processing of a protein-bound DNA end by the *E. coli* SbcCD (MR) complex. DNA Repair (Amst).

[113] Trujillo K.M., Sung P. (2001). DNA Structure-specific nuclease activities in the *Saccharomyces cerevisiae* Rad50-Mre11 complex. J Biol Chem.

[114] Langerak P., Mejia-Ramirez E., Limbo O., Russell P. (2011). Release of Ku and MRN from DNA ends by Mre11 nuclease activity and Ctp1 is required for homologous recombination repair of double-strand breaks. PLoS Genet.

[115] Chanut P., Britton S., Coates J., Jackson S.P., Calsou P. (2016). Coordinated nuclease activities counteract Ku at single-ended DNA double-strand breaks. Nat Commun.

[116] Hartsuiker E., Mizuno K., Molnar M., Kohli J., Ohta K., Carr A.M. (2009). Ctp1^CtIP^ and Rad32^Mre11^ nuclease activity are required for Rec12^Spo11^ removal, but Rec12^Spo11^ removal is dispensable for other MRN-dependent meiotic functions. Mol Cell Biol.

[117] Nakamura K., Kogame T., Oshiumi H., Shinohara A., Sumitomo Y., Agama K. (2010). Collaborative action of Brca1 and CtIP in elimination of covalent modifications from double-strand breaks to facilitate subsequent break repair. PLoS Genet.

[118] Keeney S., Giroux C.N., Kleckner N. (1997). Meiosis-specific DNA double-strand breaks are catalyzed by Spo11, a member of a widely conserved protein family. Cell.

[119] Keeney S., Kleckner N. (1995). Covalent protein-DNA complexes at the 5′ strand termini of meiosis-specific double-strand breaks in yeast. Proc Natl Acad Sci U S A.

[120] Moreau S., Ferguson J.R., Symington L.S. (1999). The nuclease activity of Mre11 is required for meiosis but not for mating type switching, end joining, or telomere maintenance. Mol Cell Biol.

[121] Tsubouchi H., Ogawa H. (1998). A novel *mre11* mutation impairs processing of double-strand breaks of DNA during both mitosis and meiosis. Mol Cell Biol.

[122] Nairz K., Klein F. (1997). *mre11S* – a yeast mutation that blocks double-strand-break processing and permits nonhomologous synapsis in meiosis. Genes Dev.

[123] Alani E., Padmore R., Kleckner N. (1990). Analysis of wild-type and *rad50* mutants of yeast suggests an intimate relationship between meiotic chromosome synapsis and recombination. Cell.

[124] Lee S.E., Bressan D.A., Petrini J.H.J., Haber J.E. (2002). Complementation between N-terminal *Saccharomyces cerevisiae mre11* alleles in DNA repair and telomere length maintenance. DNA Repair (Amst).

[125] Sung S., Li F., Park Y.B., Kim J.S., Kim A., Song O. (2014). DNA end recognition by the Mre11 nuclease dimer: insights into resection and repair of damaged DNA. EMBO J.

[126] Rahman S., Beikzadeh M., Canny M.D., Kaur N., Latham M.P. (2020). Mutation of conserved Mre11 residues alter protein dynamics to separate nuclease functions. J Mol Biol.

[127] Tran P.T., Erdeniz N., Dudley S., Liskay R.M. (2002). Characterization of nuclease-dependent functions of Exo1p in *Saccharomyces cerevisiae*. DNA Repair (Amst).

[128] Kao H.I., Campbell J.L., Bambara R.A. (2004). Dna2p helicase/nuclease is a tracking protein, like FEN1, for flap cleavage during okazaki fragment maturation. J Biol Chem.

[129] Genschel J., Modrich P. (2003). Mechanism of 5′-directed excision in human mismatch repair. Mol Cell.

[130] Chen X., Paudyal S.C., Chin R.-I., You Z. (2013). PCNA promotes processive DNA end resection by Exo1. Nucleic Acids Res.

[131] Paull T.T., Gellert M. (1999). Nbs1 potentiates ATP-driven DNA unwinding and endonuclease cleavage by the Mre11/Rad50 complex. Genes Dev.

[132] Chen L., Trujillo K.M., Van Komen S., Roh D.H., Krejci L., Lewis L.K. (2005). Effect of amino acid substitutions in the Rad50 ATP binding domain on DNA double strand break repair in yeast. J Biol Chem.

[133] Cannon B., Kuhnlein J., Yang S.H., Cheng A., Schindler D., Stark J.M. (2013). Visualization of local DNA unwinding by Mre11/Rad50/ Nbs1 using single-molecule FRET. Proc Natl Acad Sci U S A.

[134] Trujillo K.M., Roh D.H., Chen L., Van Komen S., Tomkinson A., Sung P. (2003). Yeast Xrs2 binds DNA and helps target Rad50 and Mre11 to DNA ends. J Biol Chem.

[135] Gobbini E., Vertemara J., Longhese M.P. (2018). Local unwinding of double-strand DNA ends by the MRX complex promotes Exo1 processing activity. Mol. Cell Oncol.

[136] van der Linden E., Sanchez H., Kinoshita E., Kanaar R., Wyman C. (2009). RAD50 and NBS1 form a stable complex functional in DNA binding and tethering. Nucleic Acids Res.

[137] Clerici M., Mantiero D., Lucchini G., Longhese M.P. (2005). The *Saccharomyces cerevisiae* Sae2 protein promotes resection and bridging of double strand break ends. J Biol Chem.

[138] Andres S.N., Williams R.S. (2017). CtIP/Ctp1/Sae2, molecular form fit for function. DNA Repair (Amst).

[139] Zhu M., Zhao H., Limbo O., Russell P. (2018). Mre11 complex links sister chromatids to promote repair of a collapsed replication fork. Proc Natl Acad Sci U S A.

[140] Cahill D., Carney J.P. (2007). Dimerization of the Rad50 protein is independent of the conserved hook domain. Mutagenesis.

[141] Chen L., Trujillo K., Ramos W., Sung P., Tomkinson A.E. (2001). Promotion of Dnl4-catalyzed DNA end-joining by the Rad50/Mre11/Xrs2 and Hdf1/Hdf2 complexes. Mol Cell.

[142] Chapard C., Jones R., van Oepen T., Scheinost J.C., Nasmyth K. (2019). Sister DNA entrapment between juxtaposed Smc heads and kleisin of the Cohesin complex. Mol Cell.

[143] Tatebe H., Lim C.T., Konno H., Shiozaki K., Shinohara A., Uchihashi T. (2020). Rad50 zinc hook functions as a constitutive dimerization module interchangeable with SMC hinge. Nat Commun.

[144] Li X, Tyler JK. Nucleosome disassembly during human non-homologous end joining followed by concerted HIRA- and CAF-1-dependent reassembly. Elife 2016;5. https://doi.org/10.7554/eLife.15129. 10.7554/eLife.15129 PMC4915809 27269284

[145] Tsabar M., Hicks W.M., Tsaponina O., Haber J.E. (2016). Re-establishment of nucleosome occupancy during double-strand break repair in budding yeast. DNA Repair (Amst).

[146] Hauer M.H., Gasser S.M. (2017). Chromatin and nucleosome dynamics in DNA damage and repair. Genes Dev.

[147] Murayama Y., Samora C., Kurokawa Y., Iwasaki H., Uhlmann F. (2018). Establishment of DNA-DNA interactions by the cohesin ring. Cell.

[148] Murayama Y., Uhlmann F. (2015). DNA entry into and exit out of the cohesin ring by an interlocking gate mechanism. Cell.

[149] Huber R., Kulemzina I., Ang K., Chavda A., Suranthran S., Teh J. (2016). Impairing cohesin Smc1/3 head engagement compensates for the lack of Eco1 function. Structure.

[150] Li S., Yue Z., Tanaka T. (2017). Smc3 deacetylation by Hos1 facilitates efficient dissolution of sister chromatid cohesion during early anaphase. Mol Cell.

[151] Davidson IF, Goetz D, Zaczek MP, Molodtsov MI, Huis in ’t Veld PJ, Weissmann F, et al. Rapid movement and transcriptional re‐localization of human cohesin on DNA. EMBO J 2016;35:2671–2685. https://doi.org/10.15252/embj.201695402. 10.15252/embj.201695402 PMC5167347 27799150

[152] Stigler J., Çamdere G., Koshland D., Greene E. (2016). Single-molecule imaging reveals a collapsed conformational state for DNA-bound cohesin. Cell Rep.

[153] Gligoris T., Scheinost J., Bürmann F., Petela N., Chan N., Uluocak P. (2014). Closing the cohesin ring: structure and function of its Smc3-kleisin interface. Science.

[154] Vazquez Nunez R., Ruiz Avila L.B., Gruber S. (2019). Transient DNA occupancy of the SMC interarm space in prokaryotic condensin. Mol Cell.

[155] Bürmann F., Lee B., Than T., Sinn L., O’Reilly F., Yatskevich S. (2019). A folded conformation of MukBEF and cohesin. Nat Struct Mol Biol.

[156] Diebold-Durand M., Lee H., Avila L., Noh H., Shin H., Im H. (2017). Structure of full-length SMC and rearrangements required for chromosome organization. Mol Cell.

[157] Kulemzina I., Ang K., Zhao X., Teh J., Verma V., Suranthran S. (2016). A reversible association between Smc coiled coils is regulated by lysine acetylation and is required for cohesin association with the DNA. Mol Cell.

[158] Trenner A, Sartori AA. Harnessing DNA double-strand break repair for cancer treatment. Front Oncol 2019;9. https://doi.org/10.3389/fonc.2019.01388. 10.3389/fonc.2019.01388 PMC6921965 31921645

[159] Brown J.S., O'Carrigan B., Jackson S.P., Yap T.A. (2017). Targeting DNA Repair in Cancer: Beyond PARP Inhibitors. Cancer Discov.

