# Supplementary material for: Functional and structural insights into the MRX/MRN complex, a key player in recognition and repair of DNA double-strand breaks
Source: Comput Struct Biotechnol J. 2020 May 16;18:1137–52. doi: 10.1016/j.csbj.2020.05.013 (PMC7260605; doi:10.1016/j.csbj.2020.05.013)
Supplement: Supplementary data 1 [file mmc1.pdf]

Supplementary Information

Table S1. Mutations cited in the text.

Allele	Mutation	Protein	Organism	Phenotype	Ref.
rad50-48	S679R, P682R	Rad50	*H. sapiens*	defect in Rad50 dimerization	[1]
	R805E	Rad50	*P. furiosus*	slow ATP hydrolysis	[2]
	L828F	Rad50	*P. furiosus*	defect in ATM activation	[3]
	D829N	Rad50	*P. furiosus*	defect in ATM activation	[3]
	A78T	Rad50	*S. cerevisiae*	defect in Tel1 activation	[4]
	E115K	Rad50	*E. coli*	reduced endo/exonuclease and altered DNA binding activity	[5]
rad50S	K6E	Rad50	*S. cerevisiae*	defective in removing Spo11 from DSB end	[6]
rad50S	R20M	Rad50	*S. cerevisiae*	defective in removing Spo11 from DSB end	[6]
rad50S	K81I	Rad50	*S. cerevisiae*	defective in removing Spo11 from DSB end	[6]
	Y328A	Mre11	*S. cerevisiae*	DNA damage sensitivity	[7]
	N113S	Mre11	*S. pombe*	defective Mre11 interaction with Nbs1	[8]
	S499P	Mre11	*S. cerevisiae*	defective Mre11-Rad50 interaction	[4]
	K149E	Mre11	*E. coli*	reduced endo/exonuclease and altered DNA binding activity	[5]
mre11S	T188I	Mre11	*S. cerevisiae*	defective Spo11 removal from DSB end	[9]
	R184A	Mre11	*S. cerevisiae*	DNA damage sensitive in *sgs1*Δ background	[10]
	H125N	Mre11	*S. cerevisiae*	nuclease deficient	[11]
	H52S	Mre11	*P. furiosus*	loss of exonuclease activity	[12]
	Y187C	Mre11	*P. furiosus*	loss of exonuclease activity	[13]
	R10T	Mre11	*S. cerevisiae*	*sae2*Δ DNA damage and resection defect rescue	[14]

Supplementary Figure S1

A

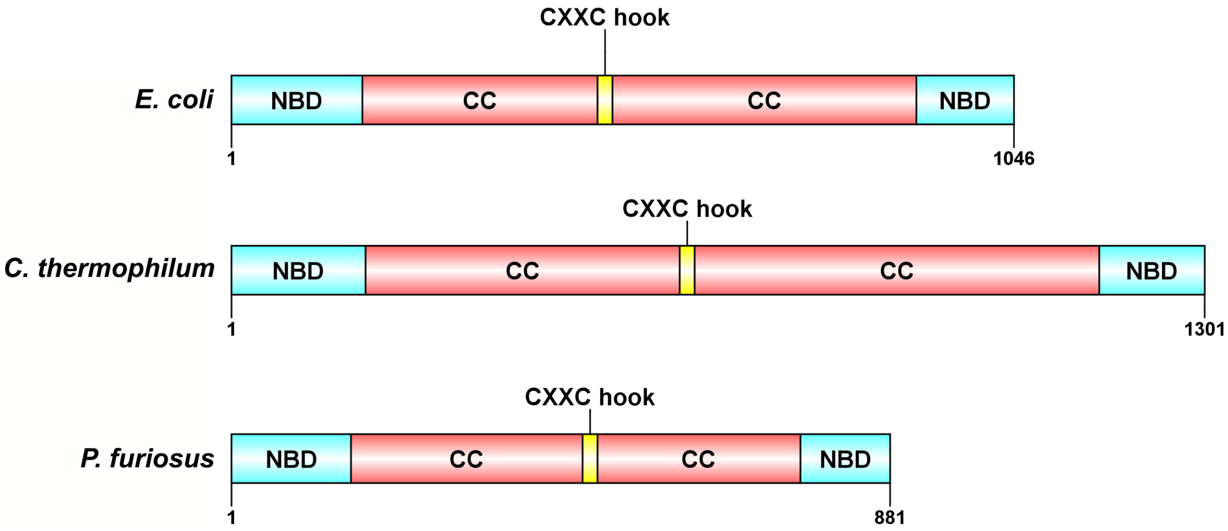

B

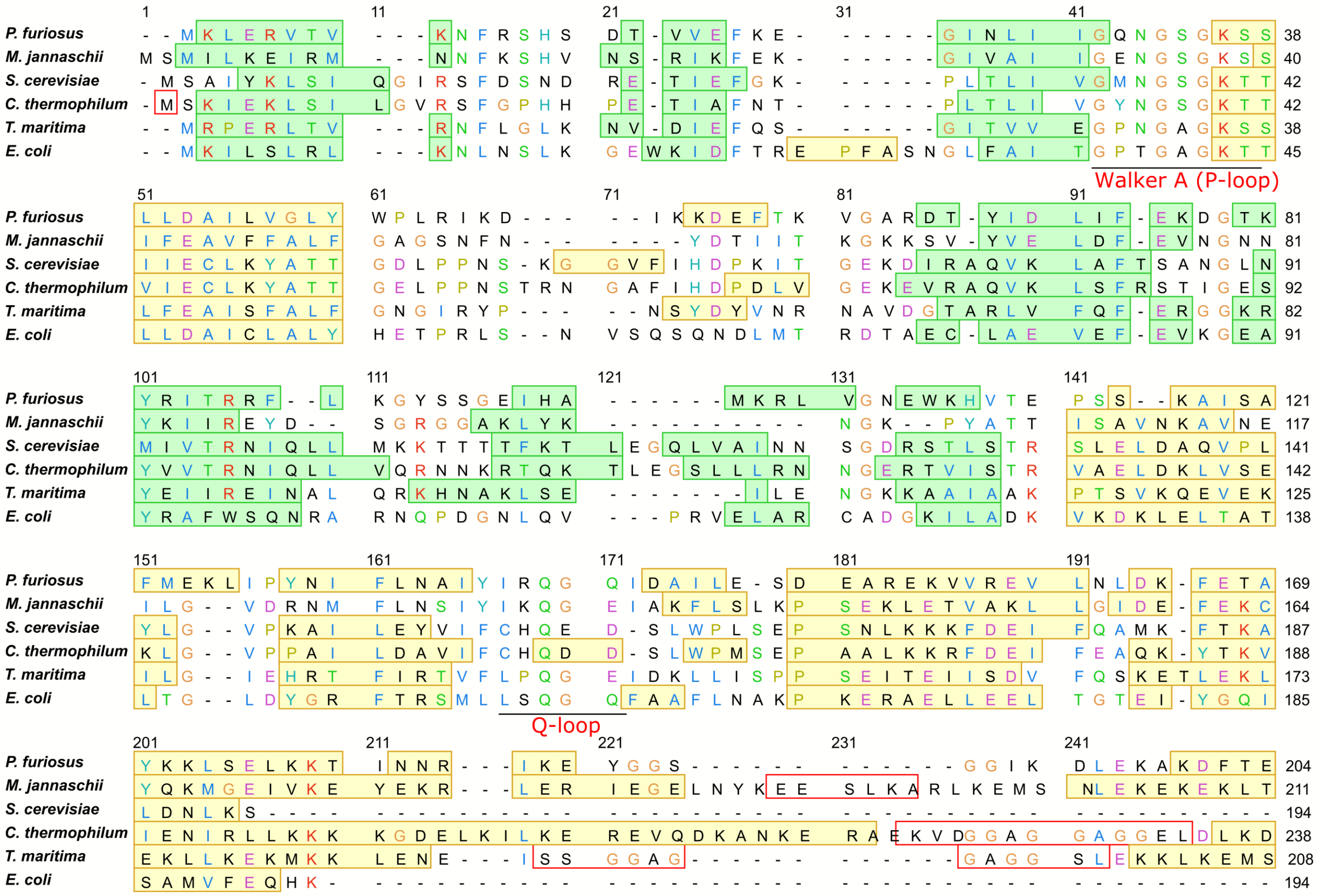

Supplementary Figure S1

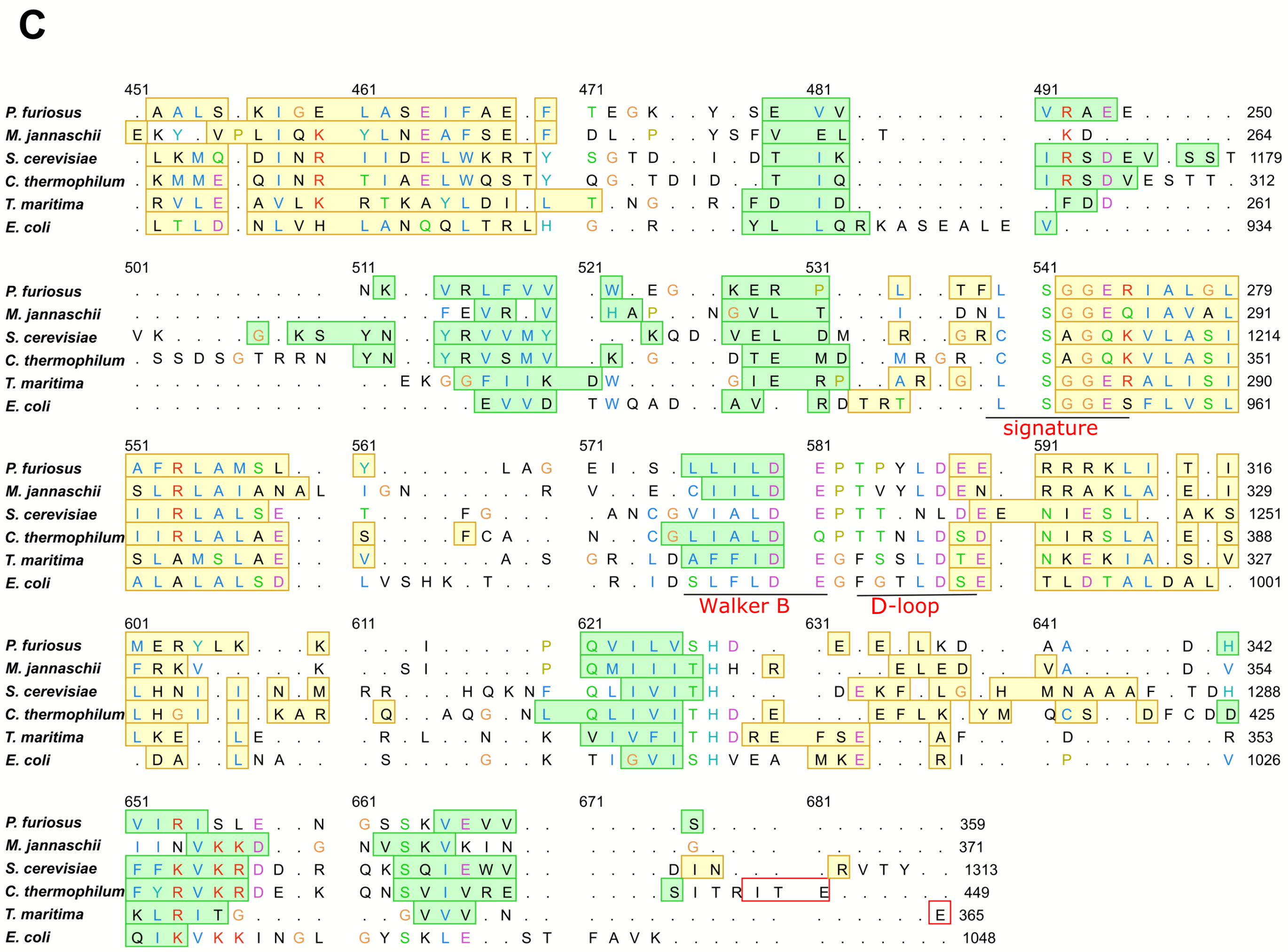

Supplementary Figure S2

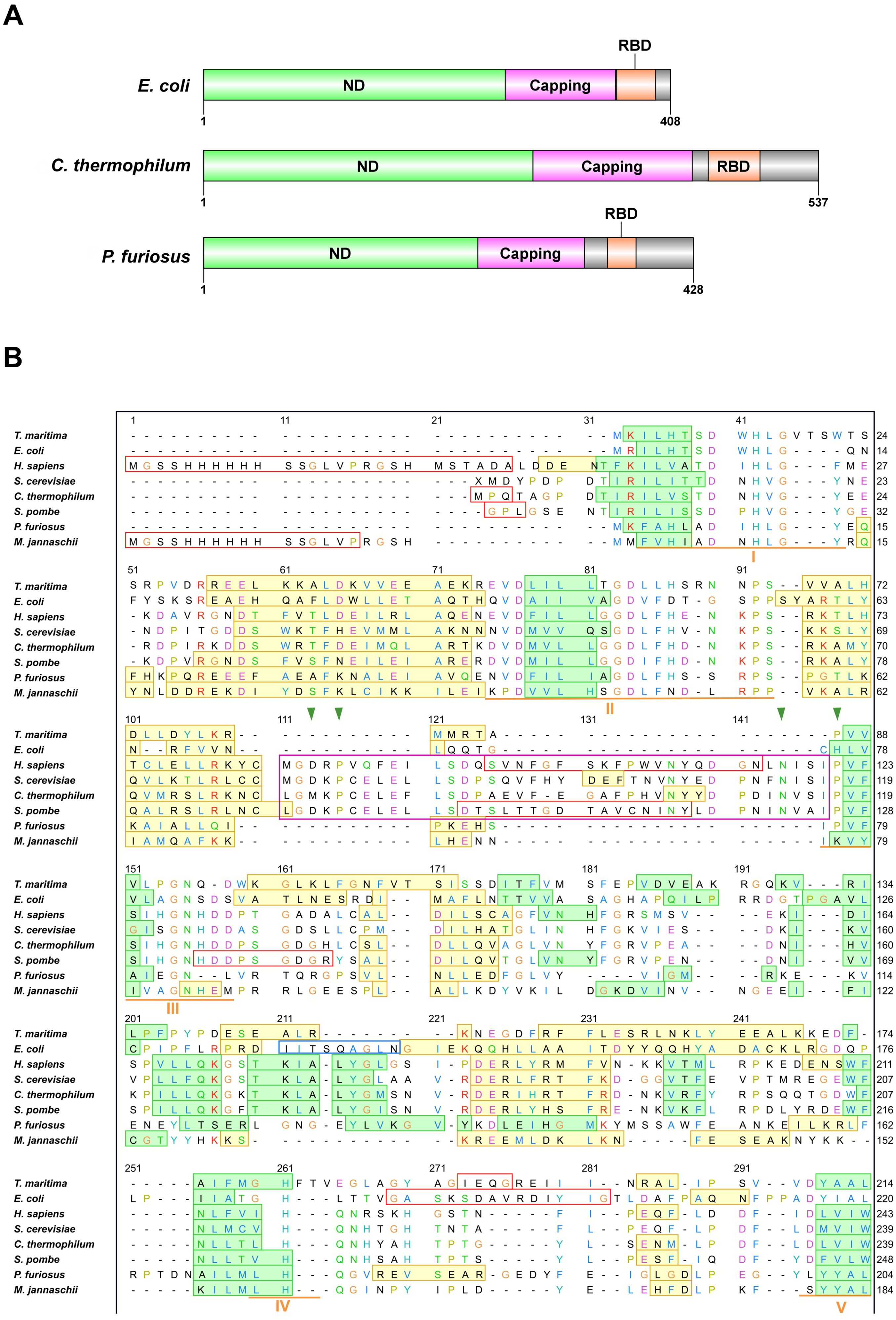

B - continued

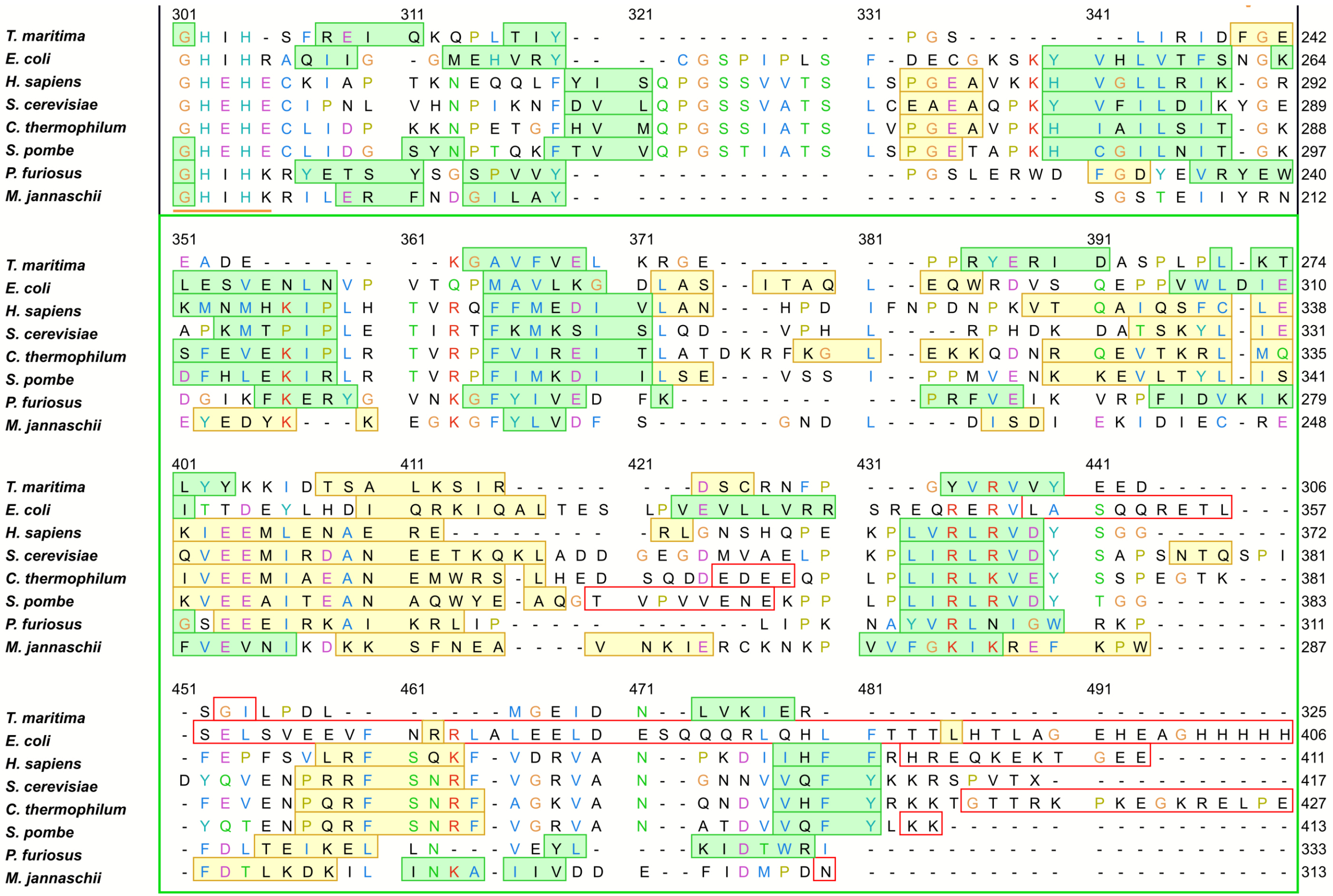
T. maritima
E. coli
H. sapiens
S. cerevisiae
C. thermophilum
S. pombe
P. furiosus
M. jannaschii

Supplementary Figure S3

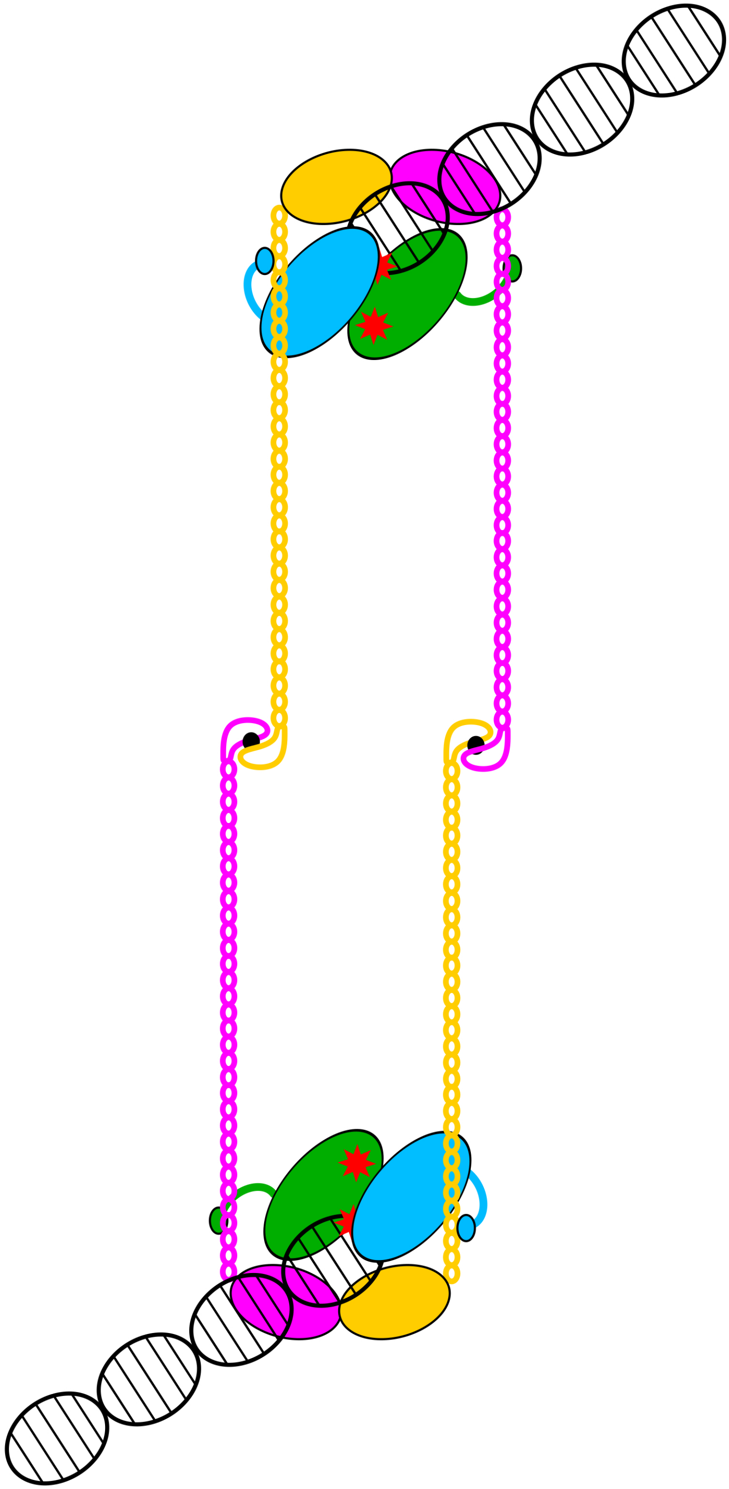

Supplementary figure legends

Supplementary Figure S1. Structural features and structural alignment of Rad50 orthologs. (A) Scheme depicting the conserved domains present in Rad50 orthologues from the indicated organisms. (B,C) Structural alignment of the lobe I (B) and lobe II (C) of Rad50 orthologues from eubacteria *T. maritima* (PDB ID: 4W9M) and *E. coli* (PDB ID: 6S6V), eukaryotes *S. cerevisiae* (model from reference [4]) and *C. thermophilum* (PDB ID:5DAC), and archaea *P. furiosus* (PDB ID:3QKU) and *M. jannaschii* (PDB ID:3AV0). Secondary structures are indicated, wherever the structure is available (otherwise the sequence is marked by a red rectangle), as follows: solid yellow rectangles, alpha helix; solid green rectangles, beta sheets. Alignment residues coloring is according to ClustalW style. Conserved functionally relevant motifs are underlined and labelled.

Supplementary Figure S2. Structural features and structural alignment of Mre11 orthologs. A. Scheme depicting the conserved domains present in Mre11 orthologs from the indicated organisms. (B) Structural alignment of the N-terminal regions of Mre11 orthologues from eubacteria *T. maritima* (PDB ID: 4NZV) and *E. coli* (PDB ID: 6S6V), eukaryotes *S. cerevisiae* (model from reference [15]), *H. sapiens* (PDB ID:3T1I), *C. thermophilum* (PDB ID:4KYE), and *S. pombe* (PDB ID:4FCX), and archaea *P. furiosus* (PDB ID:1II7) and *M. jannaschii* (PDB ID:3AV0). Secondary structures are indicated, wherever the structure is available (otherwise the sequence is marked by a red rectangle), as follows: solid yellow rectangles, alpha helix; solid green rectangles, beta sheets. Alignment residues coloring is according to ClustalW style. Functional regions of the proteins are indicated by open boxes: black box, endonuclease domain; green box, capping domain; purple box, latching loop; blue box, fastener loop. Orange bars highlight conserved phosphoesterase signature motifs (I to V). Green triangles indicate the residues identified for Nbs1 binding in *S. pombe* [8].

Supplementary Figure S3. DNA tethering can be achieved by bridging the DSB ends through an intercomplex dimerization at the Zn-hook interface of two distinct MR complexes.

References

[1] Park YB, Hohl M, Padjasek M, Jeong E, Jin KS, Kręzel A, et al. Eukaryotic Rad50 functions as a rod-shaped dimer. Nat Struct Mol Biol 2017;24:248–57. https://doi.org/10.1038/nsmb.3369.

[2] Deshpande RA, Williams GJ, Limbo O, Williams RS, Kuhnlein J, Lee J-H, et al. ATP-driven Rad50 conformations regulate DNA tethering, end resection, and ATM checkpoint signaling. EMBO J 2014;33:482–500. https://doi.org/10.1002/embj.201386100.

[3] Boswell ZK, Canny MD, Buschmann TA, Sang J, Latham MP. Adjacent mutations in the archaeal Rad50 ABC ATPase D-loop disrupt allosteric regulation of ATP hydrolysis through different mechanisms. Nucleic Acids Res 2020;48:2457–72. https://doi.org/10.1093/nar/gkz1228.

[4] Cassani C, Vertemara J, Bassani M, Marsella A, Tisi R, Zampella G, et al. The ATP-bound conformation of the Mre11-Rad50 complex is essential for Tel1/ATM activation. Nucleic Acids Res 2019;47:3550–67. https://doi.org/10.1093/nar/gkz038.

[5] Käshammer L, Saathoff JH, Lammens K, Gut F, Bartho J, Alt A, et al. Mechanism of DNA end sensing and processing by the Mre11-Rad50 complex. Mol Cell 2019;76:382-394.e6. https://doi.org/10.1016/j.molcel.2019.07.035.

[6] Alani E, Padmore R, Kleckner N. Analysis of wild-type and *rad50* mutants of yeast suggests an intimate relationship between meiotic chromosome synapsis and recombination. Cell 1990;61:419–36. https://doi.org/10.1016/0092-8674(90)90524-I.

[7] Lammens K, Bemeleit DJ, Möckel C, Clausing E, Schele A, Hartung S, et al. The Mre11:Rad50 structure shows an ATP-dependent molecular clamp in DNA double-strand break repair. Cell 2011;145:54–66. https://doi.org/10.1016/j.cell.2011.02.038.

[8] Schiller CB, Lammens K, Guerini I, Coordes B, Feldmann H, Schlauderer F, Möckel C, Schele A, Strässer K, Jackson SP, Hopfner KP. Structure of Mre11-Nbs1 complex yields insights into ataxia-telangiectasia-like disease mutations and DNA damage signaling. Nat Struct Mol Biol. 2012;19:693-700. https://doi.org/10.1038/nsmb.2323

[9] Nairz K, Klein F. *mre11S* - A yeast mutation that blocks double-strand-break processing and permits nonhomologous synapsis in meiosis. Genes Dev 1997;11:2272–90. https://doi.org/10.1101/gad.11.17.2272.

[10] Sung S, Li F, Park YB, Kim JS, Kim A, Song O, et al. DNA end recognition by the Mre11 nuclease dimer: insights into resection and repair of damaged DNA. EMBO J 2014;33:2422–2435. https://doi.org/10.15252/embj.201488299.

[11] Moreau S, Ferguson JR , Symington LS The nuclease activity of Mre11 is required for

meiosis but not for mating type switching, end joining, or telomere maintenance. Mol and Cell Biol 1999; 19:556-566. https://doi.org/10.1128/MCB.19.1.556.

[12] Williams RS, Moncalian G, Williams JS, Yamada Y, Limbo O, Shin DS, et al. Mre11 dimers coordinate DNA end bridging and nuclease processing in double-strand-break repair. Cell 2008;135:97–109. https://doi.org/10.1016/j.cell.2008.08.017.

[13] Hopfner KP, Karcher A, Craig L, Woo TT, Carney JP, Tainer JA. Structural biochemistry and interaction architecture of the DNA double-strand break repair Mre11 nuclease and Rad50-ATPase. Cell 2001;105:473–85. https://doi.org/10.1016/S0092-8674(01)00335-X.

[14] Gobbini E, Vertemara J, Longhese MP. Local unwinding of double-strand DNA ends by the MRX complex promotes Exo1 processing activity. Mol Cell Oncol 2018;5. https://doi.org/10.1080/23723556.2018.1511208.

[15] Cassani C, Gobbini E, Vertemara J, Wang W, Marsella A, Sung P, et al. Structurally distinct Mre11 domains mediate MRX functions in resection, end-tethering and DNA damage resistance. Nucleic Acids Res 2018;46. https://doi.org/10.1093/nar/gky086.
